# Supplementary material for: What we missed then, AI sees now: Revisiting legacy large extracellular vesicle data to reveal synergistic biomarkers for liver cancer screening
Source: JHEP Rep. 2025 Aug 6;7(11):101540. doi: 10.1016/j.jhepr.2025.101540 (PMC12538156; doi:10.1016/j.jhepr.2025.101540)
Supplement: Multimedia component 1 [file mmc1.pdf]

What we missed then, AI sees now: Revisiting legacy large extracellular vesicle data to reveal synergistic biomarkers for liver cancer screening

Arnulf G. Willms, Marcin Krawczyk, Henrike Julich-Haertel, Sabine K. Gries, Jesus M. Banales, Tudor Mocan, Angelina Klein, Sebastian Schaaf, Christoph Güsgen, Robert Schwab, Ingo G.H. Schmidt-Wolf, Veronika Lukacs-Kornek, Miroslaw T. Kornek

Table of contents

Supplementary Materials and Methods……………………………………………..……2

Fig. S1……………………………………………………………………………….....4

Fig. S2………………………………………………………………………………… 4

Table S1……………………………………………………………………………….5

Table S2……………………………………………………………………………….5

Table S3……………………………………………………………………………….5

Table S4……………………………………………………………………………….6

Table S5……………………………………………………………………………….7

Python Codes for LC vs. Non-LC…………………………………………...……...8

Supplementary Materials and Methods – HCC vs. CCA Classification

Dataset and Cohort Composition

This analysis was conducted on a previously published and annotated legacy dataset used in our earlier liver cancer biomarker study. For the present HCC vs. CCA re-analysis, only individuals labeled as “Group1: HCC” and “Group2: CCA” were included. The dataset contained flow cytometric measurements of large extracellular vesicle (EV) populations and classical serological markers, collected under ethically approved study protocols as described in the main manuscript. All samples were de-identified prior to analysis.

Feature Selection and Marker Panel Definition

The feature pool was identical to the LC vs. non-LC workflow and included both large EV-based markers and classical serological parameters. Initially, exploratory univariate ROC analyses were performed for each marker and selected combinations to assess individual diagnostic potential. The EV marker **AnnV$^+$EpCAM$^+$CD133$^+$gp38$^+$** showed the strongest discriminative power between HCC and iCCA cases. This EV marker was subsequently combined with serological parameters — specifically **AFP**, **Bilirubin**, and **CEA** — in various configurations to identify the most diagnostically robust panel.

Machine Learning Pipeline and Model Training

The final marker panel included the EV marker **AnnV$^+$EpCAM$^+$CD133$^+$gp38$^+$**, along with **AFP** and **Bilirubin**. This feature set was used to train and evaluate a **Random Forest (RF)** classifier. Input data was standardized using z-score normalization, and the EV marker was weighted 3× to emphasize its biological importance. The model was implemented in Python using scikit-learn, with the following pipeline:

- Data split: Stratified 70/30 train-test split
- Classifier: RandomForestClassifier(n_estimators=200, class_weight='balanced')
- Repeated evaluation: 10 runs with different random_state values (0–9)
- Metrics: Accuracy, precision, recall (sensitivity), F1-score, and AUROC

Statistical Analysis

No additional imputation was necessary, as only complete cases for the selected markers were included. Marker distributions were assessed using the Shapiro–Wilk test, and comparisons between HCC and iCCA were performed using **Mann–**

Whitney U tests. Model performance variability was quantified using standard deviation across 10 random stratified runs.

Supplementary Results

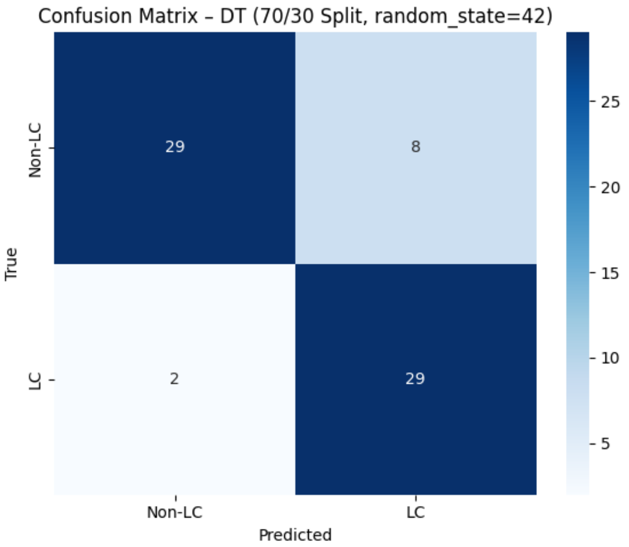

Fig. S1. Confusion matrix of the Decision Tree model (max_depth = 4, 70/30 stratified train-test split, random_state = 42) applied to the five-marker panel distinguishing between LC and non-LC patients. The matrix shows the counts of true positives (LC correctly predicted), true negatives (non-LC correctly predicted), false positives, and false negatives. The classifier achieved a sensitivity of 93.5% and a specificity of 78.4%.

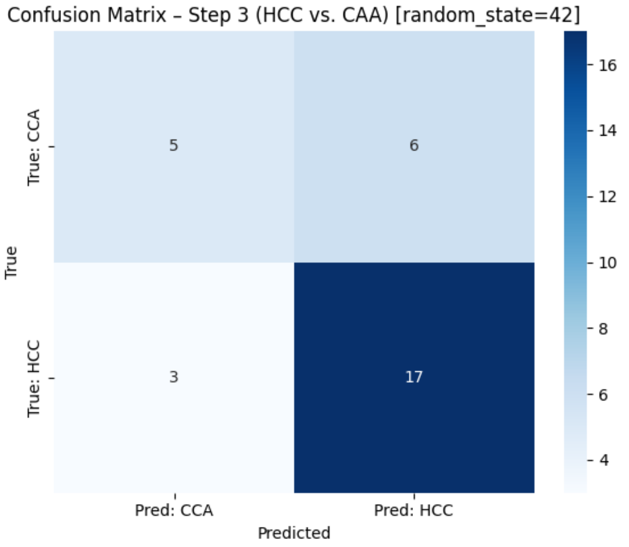

Fig. S2. Confusion matrix of the Random Forest classifier distinguishing HCC from CCA using the marker combination AFP + Bilirubin + AnnV$^+$EpCAM$^+$CD133$^+$gp38$^+$. Results are based on a stratified 70/30 train-test split (random_state=42). The model correctly classified 17 of 20 HCC cases (sensitivity: 85.0%) and 5 of 11 CCA cases (specificity: 45.5%), with an overall accuracy of 71.0%. Color intensity reflects class frequency.

Table S1. Shapiro-Wilk Normality Test for Marker Distributions

Marker	LC p-value	Non-LC p-value	LC Normal?	Non-LC Normal?
**AnnV$^+$EpCAM$^+$**	2.0422498414607304e-10	4.169339717918774e-08	No	No
**AnnV$^+$EpCAM$^+$ASGPR1$^+$**	3.0680805118699084e-12	1.4123037184177176e-09	No	No
**AnnV$^+$EpCAM$^+$CD133$^+$**	3.7788443706630876e-14	3.9833296649541804e-16	No	No
**AnnV$^+$EpCAM$^+$ASGPR1$^+$CD133$^+$**	1.107709487441404e-17	3.6967995396645924e-13	No	No
**AnnV$^+$EpCAM$^+$CD56$^+$**	4.645055007301835e-09	3.710650710309071e-12	No	No
**AnnV$^+$EpCAM$^+$ASGPR1$^+$gp38$^+$**	3.026721392873725e-20	4.394866429185583e-18	No	No
**AnnV$^+$EpCAM$^+$ASGPR1$^+$gp38$^+$CD133$^+$**	6.876331106403152e-20	2.166946180647443e-19	No	No
**AnnV$^+$EpCAM$^+$gp38$^+$**	1.502857719242987e-18	4.953815281990632e-17	No	No
**AnnV$^+$EpCAM$^+$CD133$^+$gp38$^+$**	2.0880691842653186e-18	2.3345958901760285e-18	No	No
**AnnV$^+$EpCAM$^-$CD56$^+$**	0.0004233425715938	1.1854772719033235e-10	No	No
**AnnV$^+$EpCAM$^-$CD133$^+$**	5.2130597243832224e-18	9.389114885329702e-22	No	No
**AnnV$^+$EpCAM$^-$gp38$^+$**	4.046269612770215e-15	1.8682458358121323e-10	No	No
**AnnV$^+$EpCAM$^-$CD133$^+$gp38$^+$**	1.3912658895775162e-21	1.0446741395157109e-23	No	No
**AFP**	3.972371313588609e-21	2.026842205902332e-19	No	No
**CEA**	5.455321652491732e-17	5.760598230683804e-20	No	No
**CA19-9**	8.339104922205512e-22	4.505197847239439e-19	No	No
**Bilirubin**	1.396737742385436e-16	7.101633133277574e-21	No	No

Table S2. Node-specific marker thresholds and classification decisions derived from the decision tree classifier (max_depth=4, random_state=42) trained on the 70/30 train-test split. This table lists all decision nodes used by the classifier to separate liver cancer (LC) from non-LC cases based on five biomarkers. For each node, the corresponding marker, raw threshold, classification direction, predicted class, Gini impurity, number of samples, and the LC/Non-LC sample distribution are reported. Thresholds refer to z-score standardized input values; the EV marker (AnnV$^+$EpCAM$^+$CD133$^+$gp38$^+$) was weighted ×3 prior to model fitting. All EV marker values are expressed in arbitrary units (a.u.), defined as relative marker signal per 1,000 AnnV$^+$ EVs, to allow intra-assay comparability.

Marker	Node	Z-Score Cut-off	Clinical Cut-off	Unit
**AFP**	N1	-0.132	3.19188	ng/mL
**AnnV$^+$EpCAM$^+$CD133$^+$gp38$^+$**	N13	-0.235	8.4496	a.u.
**CEA**	N2	0.209	4.69712	ng/mL
**CA19-9**	N3	-0.085	102.34015	U/mL
**Bilirubin**	N4	-0.253	1.86266	mg/dL
**AFP**	N5	0.615	6.85965	ng/mL
**AnnV$^+$EpCAM$^+$CD133$^+$gp38$^+$**	N6	-0.235	8.4496	a.u.

Table S3. Diagnostic performance of individual markers in HCC vs. CCA. This table summarizes the diagnostic performance of individual markers, including extracellular vesicle (EV) populations and classical serological parameters, for distinguishing HCC from CCA. Performance metrics include AUROC, optimal thresholds based on the Youden index, sensitivity, specificity, and overall accuracy.

Marker	Best Threshold	Sensitivity	Specificity	Accuracy	F1 Score	MW p-value
**AFP**	5.39	0.701	0.861	0.757	0.79	<0.001
**CA19-9**	18.06	0.388	0.278	0.35	0.437	<0.001
**AnnV$^+$EpCAM$^+$CD133$^+$gp38$^+$**	11.597	0.418	0.333	0.388	0.471	0.013
**Bilirubin**	0.92	0.448	0.389	0.427	0.504	0.017
**AnnV$^+$EpCAM$^-$CD133$^+$gp38$^+$**	0.0	1.0	0.0	0.65	0.788	0.024

**AnnV$^+$EpCAM$^-$CD133$^+$**	3.912	0.463	0.417	0.447	0.521	0.036
**AnnV$^+$EpCAM$^+$ASGPR1$^+$gp38$^+$CD133$^+$**	6.509	0.433	0.361	0.408	0.487	0.055
**AnnV$^+$**	35281.844	0.478	0.444	0.466	0.538	0.138
**AnnV$^+$EpCAM$^+$ASGPR1$^+$**	12.797	0.552	0.583	0.563	0.622	0.145
**AnnV$^+$EpCAM$^+$CD133$^+$**	11.542	0.493	0.472	0.485	0.555	0.151
**AnnV$^+$EpCAM$^-$CD56$^+$**	231.454	0.493	0.472	0.485	0.555	0.159
**AnnV$^+$EpCAM$^+$CD56$^+$**	13.211	0.552	0.583	0.563	0.622	0.163
**AnnV$^+$EpCAM$^+$ASGPR1$^+$CD133$^+$**	27.668	0.478	0.444	0.466	0.538	0.356
**AnnV$^+$EpCAM$^+$ASGPR1$^+$gp38$^+$**	0.0	1.0	0.0	0.65	0.788	0.502
**AnnV$^+$EpCAM$^-$gp38$^+$**	7.273	0.522	0.528	0.524	0.588	0.511
**CEA**	2.44	0.567	0.472	0.534	0.613	0.531
**AnnV$^+$EpCAM$^+$gp38$^+$**	0.0	1.0	0.0	0.65	0.788	0.641
**ALT**	42.0	0.537	0.444	0.505	0.585	0.727
**AnnV$^+$EpCAM$^+$**	96.439	0.522	0.528	0.524	0.588	0.865

Table S4. Sorted Final Model Comparison Table for HCC vs. CCA. This table summarizes the performance metrics for the top marker combinations used in the Random Forest model. It includes large EV populations and serological markers, offering insights into how various combinations influence classification metrics.

EV Marker	Serological Markers	Model	Accuracy	Precision	Recall	F1-Score	AUROC
**AnnV$^+$EpCAM$^+$CD133$^+$gp38$^+$**	AFP, Bilirubin	Random Forest	0.71	0.739	0.85	0.791	0.841
**AnnV$^+$EpCAM$^+$CD133$^+$gp38$^+$**	AFP	Random Forest	0.806	0.818	0.9	0.857	0.832
**AnnV$^+$EpCAM$^+$CD133$^+$gp38$^+$**	AFP, Bilirubin, CEA	Random Forest	0.742	0.773	0.85	0.81	0.827
**AnnV$^+$EpCAM$^+$CD133$^+$gp38$^+$**	AFP, CA19-9, Bilirubin, CEA, ALT	Random Forest	0.71	0.72	0.9	0.8	0.827
**AnnV$^+$EpCAM$^+$CD133$^+$gp38$^+$**	AFP, Bilirubin, ALT	Random Forest	0.645	0.696	0.8	0.744	0.823
**AnnV$^+$EpCAM$^+$CD133$^+$gp38$^+$**	AFP, Bilirubin, CEA, ALT	Random Forest	0.71	0.739	0.85	0.791	0.814
**AnnV$^+$EpCAM$^+$CD133$^+$gp38$^+$**	AFP, CEA	Random Forest	0.677	0.727	0.8	0.762	0.807
**AnnV$^+$EpCAM$^+$CD133$^+$gp38$^+$**	AFP, CA19-9, Bilirubin, ALT	Random Forest	0.71	0.72	0.9	0.8	0.805
**AnnV$^+$EpCAM$^+$CD133$^+$gp38$^+$**	CA19-9, Bilirubin, CEA, ALT	Random Forest	0.677	0.727	0.8	0.762	0.805
**AnnV$^+$EpCAM$^+$CD133$^+$gp38$^+$**	AFP, CA19-9, CEA	Random Forest	0.71	0.739	0.85	0.791	0.802
**AnnV$^+$EpCAM$^+$CD133$^+$gp38$^+$**	AFP, CA19-9, Bilirubin, CEA	Random Forest	0.677	0.708	0.85	0.773	0.8
**AnnV$^+$EpCAM$^+$CD133$^+$gp38$^+$**	AFP, CA19-9, Bilirubin	Random Forest	0.677	0.708	0.85	0.773	0.795

**AnnV⁺EpCAM⁺CD133⁺gp38⁺**	AFP, CA19-9, ALT	Random Forest	0.742	0.731	0.95	0.826	0.791
**AnnV⁺EpCAM⁺CD133⁺gp38⁺**	AFP, CA19-9	Random Forest	0.774	0.76	0.95	0.844	0.78
**AnnV⁺EpCAM⁺CD133⁺gp38⁺**	CA19-9, Bilirubin, CEA	Random Forest	0.677	0.727	0.8	0.762	0.777
**AnnV⁺EpCAM⁺CD133⁺gp38⁺**	AFP, CA19-9, CEA, ALT	Random Forest	0.677	0.708	0.85	0.773	0.768
**AnnV⁺EpCAM⁺CD133⁺gp38⁺**	AFP, CEA, ALT	Random Forest	0.742	0.8	0.8	0.8	0.768
**AnnV⁺EpCAM⁺CD133⁺gp38⁺**	CA19-9, Bilirubin, ALT	Random Forest	0.677	0.727	0.8	0.762	0.761
**AnnV⁺EpCAM⁺CD133⁺gp38⁺**	AFP, ALT	Random Forest	0.645	0.696	0.8	0.744	0.759
**AnnV⁺EpCAM⁺CD133⁺gp38⁺**	CA19-9, CEA, ALT	Random Forest	0.645	0.696	0.8	0.744	0.748

Table S5. Final Model Random Forest Modeling for HCC vs. CCA Detection Performance of the Random Forest (RF) model over a 70/30 train-test split for distinguishing HCC from CCA using the marker combination AFP + Bilirubin + AnnV⁺EpCAM⁺CD133⁺gp38⁺. Results are derived from a single run with random_state=42.

Metric	Accuracy	±SD	Precision	±SD	Recall	±SD	F1-Score	±SD
**70/30**	0.61	0.070	0.739	0.080	0.850	0.046	0.791	0.045

Python Codes for LC vs. Non-LC

Python Random Forest Code LC vs. Non-LC

```
# === IMPORT LIBRARIES ===
import pandas as pd
import numpy as np
from sklearn.ensemble import RandomForestClassifier
from sklearn.model_selection import train_test_split
from sklearn.metrics import (
    accuracy_score, precision_score, recall_score, f1_score,
    classification_report, confusion_matrix
)
```

```
from sklearn.preprocessing import StandardScaler
import seaborn as sns
import matplotlib.pyplot as plt

# === 1. LOAD AND PREPARE DATA ===
df = pd.read_csv("Data_4_Group4_Filled_From_Group3_FIXED_Final.csv")
print("Sample Distribution:", df["LC_label"].value_counts().to_dict())

# Define feature groups
ev_markers = ["EpCAM/CD133/gp38"]
serological_markers = ["AFP", "CEA", "CA19-9", "Bilirubin"]
all_markers = ev_markers + serological_markers

df["LC_label"] = df["LC_label"].astype(int)
X = df[all_markers].copy()
y = df["LC_label"]

# Handle missing values
if X.isnull().sum().sum() > 0:
    print("Warning: Missing values detected. Filling with median.")
    X.fillna(X.median(), inplace=True)

# === 2. APPLY FEATURE WEIGHTING ===
ev_weight = 3.0
serum_weight = 1.0
X[ev_markers] *= ev_weight
X[serological_markers] *= serum_weight

scaler = StandardScaler()
X_scaled = scaler.fit_transform(X)

# === 3. REPEATED EVALUATION (10× SPLITS) ===
acc2, prec2, rec2, f12 = [], [], [], []

for i in range(10):
    X_train, X_test, y_train, y_test = train_test_split(
        X_scaled, y, test_size=0.3, stratify=y, random_state=i
    )
    model = RandomForestClassifier(n_estimators=200, class_weight="balanced",
random_state=i)
    model.fit(X_train, y_train)
    y_pred = model.predict(X_test)

    acc2.append(accuracy_score(y_test, y_pred))
    prec2.append(precision_score(y_test, y_pred))
    rec2.append(recall_score(y_test, y_pred))
    f12.append(f1_score(y_test, y_pred))

print("\n=== Step 2 (LC vs. Non-LC) - 10× Evaluation ===")
print(f"Accuracy:  {np.mean(acc2):.4f} ± {np.std(acc2):.4f}")
print(f"Precision: {np.mean(prec2):.4f} ± {np.std(prec2):.4f}")
print(f"Recall:    {np.mean(rec2):.4f} ± {np.std(rec2):.4f}")
print(f"F1-score:  {np.mean(f12):.4f} ± {np.std(f12):.4f}")

# === 4. SINGLE RUN VISUALIZATION (random_state=42) ===
print("\n=== Single-Run Visualization for random_state=42 ===")
X_train, X_test, y_train, y_test = train_test_split(
    X_scaled, y, test_size=0.3, stratify=y, random_state=42
)
vis_model = RandomForestClassifier(n_estimators=200, class_weight="balanced",
random_state=42)
vis_model.fit(X_train, y_train)
```

```
y_pred_vis = vis_model.predict(X_test)

print("\nClassification Report (random_state=42):")
print(classification_report(y_test, y_pred_vis))

cm = confusion_matrix(y_test, y_pred_vis)
plt.figure(figsize=(6,5))
sns.heatmap(cm, annot=True, fmt="d", cmap="Blues",
            xticklabels=["Non-LC", "LC"], yticklabels=["Non-LC", "LC"])
plt.xlabel("Predicted")
plt.ylabel("True")
plt.title("Confusion Matrix - Step 2 (LC vs. Non-LC) [random_state=42]")
plt.tight_layout()
plt.show()
```

Python Decision Tree Code LC vs. Non-LC

```
# === IMPORT LIBRARIES ===
# === IMPORT LIBRARIES ===
import pandas as pd
import numpy as np

from sklearn.tree import DecisionTreeClassifier
from sklearn.model_selection import train_test_split, cross_val_score, StratifiedKFold
from sklearn.preprocessing import StandardScaler

# === LOAD & CLEAN DATA ===
df = pd.read_csv("Data_4_Group4_Filled_From_Group3_FIXED_Final.csv")
df = df[df["Group"] != "Group5: OTHER CANCER"]

# === DEFINE FEATURES & LABEL ===
features = ["EpCAM/CD133/gp38", "AFP", "CEA", "CA19-9", "Bilirubin"]
X = df[features].copy()
y = df["LC_label"].astype(int)

# === PREPROCESS ===
X.fillna(X.median(), inplace=True)
X["EpCAM/CD133/gp38"] *= 3.0
scaler = StandardScaler()
X_scaled = scaler.fit_transform(X)

# === EVALUATION FUNCTION ===
def evaluate_split(X_data, y_data, strategy_name):
    scores = {"Evaluation": [], "Metric": [], "Mean": [], "SD": []}
    model = DecisionTreeClassifier(max_depth=4, class_weight="balanced", random_state=42)
    cv = StratifiedKFold(n_splits=10, shuffle=True, random_state=42)

    for metric in ["accuracy", "precision", "recall", "f1"]:
        result = cross_val_score(model, X_data, y_data, cv=cv, scoring=metric)
        scores["Evaluation"].append(strategy_name)
        scores["Metric"].append("F1-score" if metric == "f1" else metric.capitalize())
        scores["Mean"].append(np.mean(result))
        scores["SD"].append(np.std(result))
    return pd.DataFrame(scores)

# === RUN FOR EACH STRATEGY ===
splits = {
    "50/50": 0.5,
    "60/40": 0.4,
    "70/30": 0.3,
```

```
    "80/20": 0.2,
    "90/10": 0.1,
    "Full Fit": 0.0,
}

all_results = []

for label, test_size in splits.items():
    if test_size > 0:
        X_train, _, y_train, _ = train_test_split(X_scaled, y, test_size=test_size,
stratify=y, random_state=42)
    else:
        X_train, y_train = X_scaled, y
    all_results.append(evaluate_split(X_train, y_train, label))

df_summary = pd.concat(all_results, ignore_index=True)

# === SPLIT DATA ===
from sklearn.model_selection import train_test_split
from sklearn.tree import DecisionTreeClassifier
from sklearn.metrics import confusion_matrix, ConfusionMatrixDisplay

X_train, X_test, y_train, y_test = train_test_split(X_scaled, y, test_size=0.3,
stratify=y, random_state=42)

# === TRAIN MODEL ===
model = DecisionTreeClassifier(max_depth=4, class_weight="balanced", random_state=42)
model.fit(X_train, y_train)

# === VISUALIZE DECISION TREE (70/30 Split) ===
from sklearn.tree import plot_tree

plt.figure(figsize=(20, 10))
plot_tree(
    model,
    feature_names=features,
    class_names=["Non-LC", "LC"],
    filled=True,
    rounded=True,
    impurity=True,
    fontsize=10
)
plt.title("Decision Tree – 70/30 Train-Test Split (random_state=42)", fontsize=16)
plt.tight_layout()
plt.savefig("DecisionTree_70_30_Split_Colored.png", dpi=300)
plt.show()


# === PREDICT & CONFUSION MATRIX ===
y_pred = model.predict(X_test)
cm = confusion_matrix(y_test, y_pred)
TN, FP, FN, TP = cm.ravel()

# === CALCULATE SENSITIVITY & SPECIFICITY ===
sensitivity = TP / (TP + FN)
specificity = TN / (TN + FP)

print("=== 70/30 Confusion Matrix Metrics ===")
print(f"Sensitivity (Recall):  {sensitivity:.3f}")
print(f"Specificity:           {specificity:.3f}")

# === PLOT CONFUSION MATRIX ===
```

```
import matplotlib.pyplot as plt
import seaborn as sns

plt.figure(figsize=(6,5))
sns.heatmap(cm, annot=True, fmt="d", cmap="Blues",
            xticklabels=["Non-LC", "LC"], yticklabels=["Non-LC", "LC"])
plt.xlabel("Predicted")
plt.ylabel("True")v
plt.title("Confusion Matrix – DT (70/30 Split, random_state=42)")
plt.tight_layout()
plt.show()

from sklearn.metrics import roc_auc_score

# === GET PROBABILITIES FOR AUROC ===
y_proba = model.predict_proba(X_test)[:, 1]  # Probabilities for the positive class

# === CALCULATE AUROC ===

auroc = roc_auc_score(y_test, y_proba)

print(f"AUROC (70/30 Split):   {auroc:.3f}")

# === EXPORT TO CSV (OPTIONAL) ===
df_summary.to_csv("DT_10xCV_Transparency_Table.csv", index=False)

# === DISPLAY RESULTS ===
df_summary
```
