# Supplementary material for: What we missed then, AI sees now: Revisiting legacy large extracellular vesicle data to reveal synergistic biomarkers for liver cancer screening
Source: JHEP Rep. 2025 Aug 6;7(11):101540. doi: 10.1016/j.jhepr.2025.101540 (PMC12538156; doi:10.1016/j.jhepr.2025.101540)
Supplement: Multimedia component 4 [file mmc4.pdf]

Research article

JHEP|Reports

What we missed then, AI sees now: Revisiting legacy large extracellular vesicle data to reveal synergistic biomarkers for liver cancer screening

Authors

Arnulf G. Willms, Marcin Krawczyk, Henrike Julich-Haertel, …, Robert Schwab, Ingo G.H. Schmidt-Wolf, Veronika Lukacs-Kornek[†]Miroslaw T. Kornek[†]

Correspondence

miroslawkornek@web.de (M.T. Kornek), vlukacsk@uni-bonn.de (V. Lukacs-Kornek).

Graphical abstract

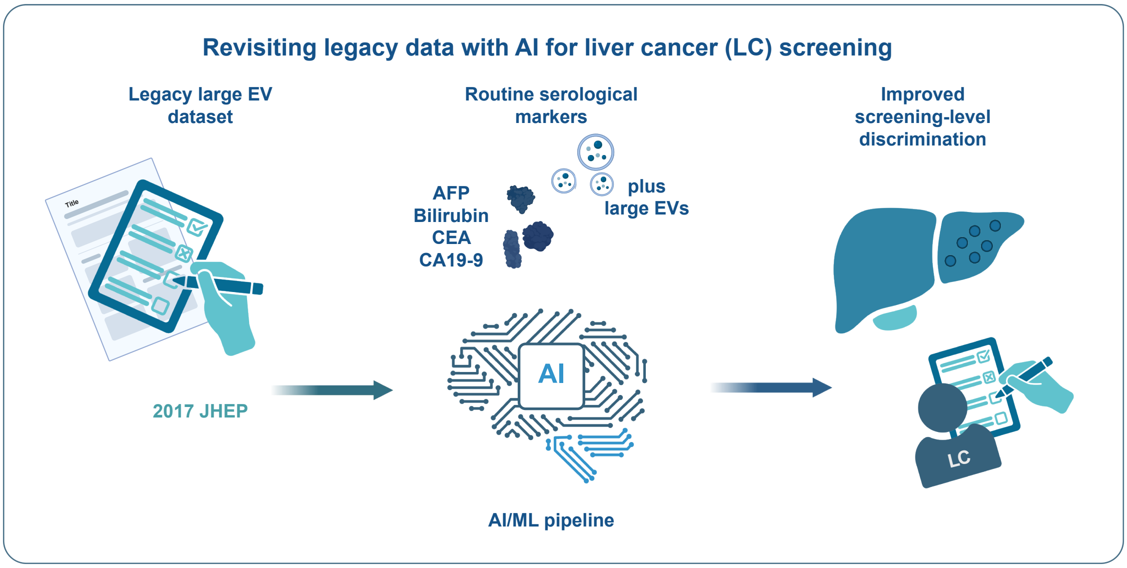

Highlights:

- We re-analyzed a legacy EV dataset (*Journal of Hepatology*, 2017) using modern AI-assisted classification models.
- A rare EV subset (AnnV$^+$EpCAM$^+$CD133$^+$gp38$^+$) showed the strongest discriminative power for liver cancer detection.
- Both Random Forest and Decision Tree classifiers achieved high diagnostic accuracy, exceeding traditional serological markers.
- The interpretable Decision Tree model offers a clinically transparent diagnostic tool applicable at the bedside.
- Our study underscores the value of AI tools for re-mining legacy biomarker data and promoting data-driven precision diagnostics.

Impact and implications:

This study revisits a legacy extracellular vesicle dataset using modern artificial intelligence-assisted analysis to identify synergistic biomarker combinations for liver cancer screening. The results demonstrate that archived data, when re-analyzed with advanced computational tools, can yield novel and clinically relevant insights – particularly for screening liver malignancies. This approach may serve as a reproducible and transparent blueprint for similar efforts in liver diseases, oncology, and biomedical research more broadly. These findings are relevant to clinicians, translational researchers, and policymakers aiming to advance precision screening and early detection strategies. More broadly, this work underscores the role of artificial intelligence as a scientific collaborator and offers a model for responsible, sustainable innovation in biomedical research.

https://doi.org/10.1016/j.jhepr.2025.101540
© 2025 The Authors. Published by Elsevier B.V. on behalf of European Association for the Study of the Liver (EASL). This is an open access article under the CC BY license (http://creativecommons.org/licenses/by/4.0/).

What we missed then, AI sees now: Revisiting legacy large extracellular vesicle data to reveal synergistic biomarkers for liver cancer screening

Arnulf G. Willms[1], Marcin Krawczyk[2,3,4], Henrike Julich-Haertel[2], Sabine K. Gries[2], Jesus M. Banales[5,6], Tudor Mocan[7], Angelina Klein[1], Sebastian Schaaf[1], Christoph Güsgen[1], Robert Schwab[1], Ingo G.H. Schmidt-Wolf[8], Veronika Lukacs-Kornek[9,†,*], Miroslaw T. Kornek[1,8,†,*]

JHEP Reports **2025**. vol. 7 | 1–11

Check for updates

Background & Aims: In our 2017 publication, we introduced a novel concept for liver cancer detection using large extracellular vesicles (EVs), specifically AnnV$^+$EpCAM$^+$ASGPR1$^+$ tumor-associated microparticles. Despite promising biology, the diagnostic utility was limited by the analytical tools available at the time, resulting in modest performance (AUC 0.70, 75% sensitivity, 47% specificity).

Methods: In the present study, we revisited this legacy dataset – now supplemented with unpublished but previously collected measurements (N = 166) – and applied modern artificial intelligence-assisted analytical strategies. We evaluated a wide range of combinatorial models using Random Forest and Decision Tree classifiers, incorporating both rare large EV populations and classical serological markers (AFP, CEA, CA19-9, bilirubin).

Results: The RF model combining AnnV$^+$EpCAM$^+$CD133$^+$gp38$^+$ large EVs with classical serological markers achieved a mean accuracy of 88.2%, recall of 91.6%, and F1-score of 87.0% across 10 stratified train-test runs. This substantially outperformed earlier analysis efforts. To support clinical translation, we additionally developed a simplified decision tree model based on the same marker inputs, offering a visual and rule-based alternative that remained robust, with an average accuracy of 86.6% and recall of 87.3% and a sensitivity of 94% and a specificity of 78% (70/30 split) across 10 stratified train-test runs.

Conclusions: This study demonstrates how legacy data, when re-analyzed with artificial intelligence-supported tools, can reveal clinically actionable insight. Large EVs – particularly derived from rare progenitor-like subpopulations – combined with classical serum markers, provide a promising non-invasive screening approach.

© 2025 The Authors. Published by Elsevier B.V. on behalf of European Association for the Study of the Liver (EASL). This is an open access article under the CC BY license (http://creativecommons.org/licenses/by/4.0/).

Introduction

Biomedical research and research in general often advance not only through the generation of new data, but through the re-examination of existing with novel analytical tools and knowledge. Here, in this work, we revisit a previously published dataset that originally appeared in the *Journal of Hepatology* in 2017, where a novel concept was introduced: the use of large, Annexin V (AnnV)-positive extracellular vesicles (EVs), also known as microparticles at that time or ectosomes or microvesicles, as potential biomarkers in liver cancer (LC).[1] Although biologically intriguing, that early analysis yielded modest diagnostic performance and relevance and the study remained conceptually ahead of its time. Today, empowered by artificial intelligence (AI) tools, advanced cut-off optimization, and multi-dimensional pattern recognition, previously some 'underperforming' data can be re-analyzed with vastly improved fidelity.[2] We aim to demonstrate how a legacy dataset, originally limited by the analytical tools of its era, can – through AI-assisted reprocessing – yield a clinically viable and interpretable screening tool for LC. This study does not rely on new patient data, we take legacy data, published and unpublished, and exemplifies how AI-driven analytics can exploit novel insight that we did not discover almost a decade ago.

In our 2017 publication, we introduced a conceptual framework for using large EVs — the best-performing large EV population was specifically AnnV$^+$EpCAM$^+$ASGPR1$^+$

* Corresponding authors. Addresses: German Armed Forces Hospital Koblenz, Department of General, Visceral and Thoracic Surgery, Rübenacher Str. 170, 56072 Koblenz, Germany (M.T. Kornek); Institute of Molecular Medicine and Experimental Immunology, University Bonn, Venusberg Campus 1, Geb. 12, BMZII, 53127 Bonn, Germany (V. Lukacs-Kornek).
E-mail addresses: miroslawkornek@web.de (M.T. Kornek), vlukacsk@uni-bonn.de (V. Lukacs-Kornek).
† Shared last authorship.
https://doi.org/10.1016/j.jhepr.2025.101540

tumor-associated microparticles – for LC detection.[1] At that time, the approach was technologically novel, and the idea that large EVs could function as non-invasive cancer biomarkers as part of a liquid biopsy was still in its infancy. Despite the biological promise, the diagnostic performance remained modest, with an AUC of 0.70, associated with 75% sensitivity, and 47% specificity.[1] As said, the concept was ahead of its time, constrained by the limitations of manual thresholding, univariate analysis, and the absence of integrative modeling tools.

Despite the availability of screening programs for high-risk populations such as patients with cirrhosis, LC is still often diagnosed at an advanced stage, especially intrahepatic cholangiocellular carcinoma (iCCA). Current surveillance strategies typically rely on ultrasound imaging, sometimes in combination with serum α-fetoprotein (AFP) levels.[3] However, no diagnostic biomarkers are in the current AASLD and EASL guidelines recommended or FDA approved.[3,4] Some legacy markers as AFP, carcinoembryonic antigen (CEA), and CA19-9 are routinely assessed in those patients. The latter two as CEA and CA19-9 are unspecific for LC. Some new markers emerged for risk stratification as the combination of des-gamma carboxy-prothrombin (DCP) and lectin-bound AFP (AFP-L3),[5] which have insufficient sensitivity if used alone for early-stage hepatocellular carcinoma (HCC) detection.[6] According to the current AASLD guideline AFP-L3% and DCP may be complimentary to AFP.[4] Ultrasound for primary LC has limited sensitivity, particularly for early-stage tumors and iCCA, and are subject to considerable operator variability as discussed in the current EASL guidelines.[3] Moreover, many of the biological signals associated with malignant transformation overlap with chronic liver disease as hepatic fibrosis and cirrhosis, rendering it difficult to distinguish cancer from inflammatory or fibrotic processes using conventional serological markers alone.[7]

Particularly, EVs, small and large EVs, have garnered attention as a minimally invasive source of molecular information that reflects cellular activity as reviewed by us and others.[8,9] Derived from nearly all cell types, EVs are released into the circulation under both physiological and pathological conditions. Large EVs, in particular – which bud directly from the plasma membrane and can be readily detected via flow cytometry as demonstrated by us in the past – carry surface markers and internal cargo reflective of their cell of origin.[1,10,11] As such, they hold potential not only for detecting malignancy, but also for understanding the tumor microenvironment and its interface with surrounding hepatic tissue. Large EV size may vary and typically range between 100/150 nm and 1,000 nm according to the literature. Although overlapping in size with small EVs as exosomes and eventually with lipoproteins, large EVs have a unique biogenesis, budding from the cell plasma membrane, lagging an own ongoing metabolism, to keep the asymmetric vesicle membrane in place.[12,13]

Our aim was to develop AI supported a simple, interpretable, diagnostic approach that could serve as a frontline screening tool for liver integrating both large EV-based and legacy serological markers. Beyond the diagnostic metrics, this study underscores a broader principle: that legacy data, when reassessed with thoughtful algorithms and clinical grounding, can still yield discoveries that were once overlooked. This is not simply an analytical refinement – it is an exercise in scientific resurrection.

Patients and methods

Patient cohort and sample origin

The dataset used in this study originates from a previously published cohort,[1] consisting of human serum samples collected from patients diagnosed with HCC, cholangiocarcinoma (CCA), cirrhosis, and healthy controls. All samples had been previously collected under informed consent and in accordance with local ethical committee approvals. The Ethics Commissions of the State Chambers of Medicine in Rhineland-Palatinate, Germany; Saarland, Germany; San Sebastian, Spain; and Warsaw, Poland, approved the original study (approval numbers as follows: 837.151.13 (8836-F),167/11, PI2014187, KB/41/A/2016, and AKB/145/2014). No new experiments were conducted; re-analysis only of existing anonymized datasets. Patient demographics were published in the original studies.[1,10]

Large extracellular vesicle isolation and staining

Large EVs, also referred to as microvesicles, microparticles, or ectosomes, were isolated using differential centrifugation and stained using a previously established flow cytometric protocol. Full details of the isolation, storing, AnnV gating, and surface staining for gp38, EpCAM, CD133, and ASGPR1 are published in our prior work and elsewhere.[1,10] The staining of EVs for gp38 followed the optimized protocol previously validated and published in our group.[10,14,15]

Flow cytometric analysis of large EVs

EVs were analyzed on a MACSQuant10 Analyzer (Miltenyi Biotec, Bergisch-Gladbach, Germany) using high-sensitivity settings for small particle detection as described fully in our initial publication and elsewhere.[1,14,16] Only events positive for AnnV and within the defined size gate (~500–1,000 nm) were considered valid large EVs. Compensation was applied based on single-stained controls, and gating was set according used isotype controls and consistently reviewed by experienced operators. For further details please see prior publications.[1,10,14] Our large EV had been analyzed according to the ISEV MISEV guideline 2018.[10,17]

Serological marker data and imputation

Serological data for AFP, CEA, carbohydrate antigen 19-9 (CA19-9), and bilirubin were retrieved from anonymous stored legacy study data. In cases where values were missing, particularly in healthy control individuals, imputation was performed using the median values of patients with cirrhosis, assuming that cirrhotic values surpass those of healthy study precipitants. This approach reflects the clinical scenario where patients with cirrhosis represent the main differential diagnosis population for LC surveillance.

Marker panel selection and optimization

We began this re-analysis with a broad panel of large EV populations and classical serological markers previously available from the 2017 dataset. Initially, multiple EV combinations were evaluated, including $AnnV^+EpCAM^+$, $AnnV^+ASGPR1^+$, $AnnV^+CD133^+$, $AnnV^+gp38^+$, and some of their Boolean combinations in combination with the initial pool

of classical serological markers included AFP, CEA, CA19-9, bilirubin, alanine transaminase, aspartate transaminase, and gamma-glutamyl transferase. These were screened using univariate receiver operating characteristic (ROC) analysis to assess their ability to distinguish LC (LC, HCC, and CCA) from non-LC (non-cancer controls: those with cirrhosis and healthy individuals). Each marker was tested not only in isolation, but also in combination with others to identify synergistic effects.

Performance evaluation Random Forest model

For data preprocessing, missing values were imputed using median values, and serological markers were log-transformed if necessary (N = 166). EV markers were manually weighted (× 3) to reflect their biological prominence, and all features were standardized using z-score normalization. Model performance was evaluated using two complementary machine-learning approaches. First, a Random Forest (RF) classifier with 200 trees was trained and evaluated using five-fold cross-validation. To independently assess generalizability, the same model was additionally trained and tested using a 70/30 stratified train-test split. Metrics including accuracy, precision, recall (sensitivity), F1 score, and area under the receiver operating characteristic curve (AUROC) were calculated. This model provided robust classification insights and was used to compare various combinations of EV and serological markers. The model was trained on the standardized feature set and evaluated on the hold-out test set. Performance was measured using the same metrics, and a confusion matrix was generated to visualize classification accuracy and class-specific performance. Although the metrics were more conservative, this approach provided a realistic estimation of the model's diagnostic utility in unseen data.

Additionally, to assess the internal robustness of our RF model, we performed bootstrap validation with 1,000 iterations. Performance metrics including accuracy, precision, recall, and F1-score were recalculated for each resample, and 95% CIs were computed to estimate metric variability. This further validated the consistency and generalizability of our model.

External validation of the final RF model

To assess the robustness and generalizability of our final RF classifier, we applied it to an independent dataset (n = 37) derived from a second cohort processed separately.[10] Only cases with complete data across all markers ($AnnV^+EpCAM^+CD133^+gp38^+$, AFP, CEA, CA19-9) were included. The same preprocessing pipeline was used, including 3 × weighting of EV features, feature standardization, and model training. We performed 10 repeated train-test splits (70/30 stratified) to assess stability and calculated accuracy, precision, recall, and F1-score. Confusion matrix analysis was also conducted for a representative split (random_state = 9).

Decision tree classifier: construction, evaluation, and visualization

To complement the RF classifier and compute a more interpretable diagnostic model, we trained a Decision Tree (DT) classifier using the same feature set: $AnnV^+EpCAM^+CD133^+gp38^+$ large EVs, AFP, CEA, CA19-9, and bilirubin. The data were first standardized using z-transformation, and EV markers were weighted 3 × before model fitting, consistent with the RF preprocessing pipeline. The DT was trained using the DecisionTreeClassifier from scikit-learn (v1.6.1), with max_depth = 4 to balance interpretability and performance, and class_weight = 'balanced' to account for class imbalance. The resulting tree was visualized using the plot_tree function with customized color-coding to represent class purity (blue for LC, orange for non-LC), and the Gini impurity score was displayed at each node. Unlike black-box models, the DT provides transparent, rule-based classification logic, offering clinicians an interpretable pathway from biomarker input to diagnosis. This tree-based model represents a transparent and standalone diagnostic tool: each decision node corresponds to a binary rule based on a biomarker threshold, ultimately classifying patients as LC or non-LC. Performance was evaluated over 10 stratified 50/50, 60/40, 70/30, 80/20, 90/10 and full split train-test splits to assess robustness and generalizability. Full diagnostic metrics with standard deviations are provided. To further evaluate clinical applicability, we derived sensitivity, specificity, and AUROC from a representative 70/30 train-test split using a fixed random_state (42). To ensure model interpretability and support clinical translation, we extracted the decision thresholds used by the final DT classifier trained on the 70/30 train-test split (random_state = 42). The DT was trained on z-score standardized data with 3 × weighting applied to the EV marker $AnnV^+EpCAM^+CD133^+gp38^+$ to reflect biological importance. Feature values were imputed using group-specific medians. The DT model was visualized using scikit-learn's plot_tree function and evaluated using 10-fold stratified cross-validation. The resulting thresholds for each decision node were recorded and matched to known clinical reference values where available.

Statistical analysis

To assess the distribution characteristics of marker data within the training dataset, we employed the Shapiro–Wilk test for normality. Given that most markers significantly deviated from a Gaussian distribution, we proceeded with non-parametric testing. Specifically, the Mann–Whitney *U* test was used to evaluate differences in marker levels between LC and non-LC groups, as summarized in Table 1. Chi-square tests were used for categorical comparisons, where appropriate. Missing serological values in the training set were imputed using median values from patients with cirrhosis, assuming clinical comparability with high-risk screening populations. The external validation cohort included only samples with complete biomarker profiles (no imputation). To reflect the biological signal prominence of EV-derived markers, a 3 × weighting factor was applied before model training. RF classifiers were used to evaluate marker panel combinations (LC *vs.* non-LC). Models were trained with 200 estimators using five-fold cross-validation. Classification performance was assessed via accuracy, precision, recall, F1 score, and AUROC, with predictions generated using cross_val_predict. Scoring thresholds were defined using the Youden index derived from ROC curves to optimize the sensitivity-specificity balance. Marker selection was based on additive performance across

Table 1. Diagnostic performance of individual markers.

Marker	Best threshold	Sensitivity	Specificity	Accuracy	F1 score	MW *p*-value
AnnV$^+$EpCAM$^+$	134.969	0.369	0.886	0.65	0.49	0.031
AnnV$^+$EpCAM$^+$ASGPR1$^+$	14.493	0.466	0.74	0.615	0.525	0.027
AnnV$^+$EpCAM$^+$CD133$^+$	11.542	0.505	0.707	0.615	0.545	0.069
AnnV$^+$EpCAM$^+$ASGPR1$^+$CD133$^+$	38.13	0.427	0.886	0.677	0.547	0.023
AnnV$^+$EpCAM$^+$CD56$^+$	14.493	0.476	0.715	0.606	0.524	0.091
AnnV$^+$EpCAM$^+$ASGPR1$^+$gp38$^+$	5.291	0.087	0.959	0.562	0.154	0.015
AnnV$^+$EpCAM$^+$ASGPR1$^+$gp38$^+$CD133$^+$	11.737	0.359	0.894	0.65	0.484	0.007
AnnV$^+$EpCAM$^+$gp38$^+$	6.826	0.126	0.927	0.562	0.208	0.014
AnnV$^+$EpCAM$^+$CD133$^+$gp38$^+$	15.564	0.427	0.87	0.668	0.54	0.005
AnnV$^+$EpCAM$^-$CD56$^+$	346.446	0.097	0.927	0.549	0.164	0.311
AnnV$^+$EpCAM$^-$CD133$^+$	7.03	0.456	0.724	0.602	0.511	0.304
AnnV$^+$EpCAM$^-$gp38$^+$	21.739	0.282	0.854	0.593	0.387	0.293
AnnV$^+$EpCAM$^-$CD133$^+$gp38$^+$	7.576	0.136	0.935	0.571	0.224	0.267
AFP	6.43	0.466	0.902	0.704	0.589	0.442
CEA	2.86	0.417	0.894	0.677	0.541	0.321
CA19-9	17.37	0.524	0.894	0.726	0.635	0.207
Bilirubin	6.6	0.146	0.951	0.584	0.242	0.257

Diagnostic performance of individual markers, including EV populations and classical serological parameters, for differentiating liver cancer (LC) from non-LC. Performance metrics include AUROC, optimal cut-offs based on Youden index, sensitivity, specificity, and accuracy. Statistical comparisons between LC and non-LC groups were performed using the two-sided Mann–Whitney *U* test. Normality of marker distributions was assessed using the Shapiro–Wilk test. AFP, α-fetoprotein; AnnV, Annexin V; CA19-9, carbohydrate antigen 19-9; CEA, carcinoembryonic antigen.

multiple combinations rather than feature elimination techniques such as least absolute shrinkage and selection operator (LASSO) or principal component analysis (PCA). In addition to mean and standard deviation, 95% CIs were computed using bootstrap validation (1,000 iterations) to assess the internal robustness of performance metrics. Stratified sampling was consistently applied during 10 × 70/30 train-test splits to ensure class balance across all evaluations.

For external validation, the final RF model was applied to an independent, previously untrained dataset with complete biomarker profiles. The same preprocessing pipeline was used, including z-score standardization and 3 × weighting of EV markers. Model performance was evaluated over 10 repeated stratified 70/30 train-test splits. Classification accuracy, precision, recall, and F1-score were averaged, and a representative confusion matrix from a fixed split (random_state = 9) was used to illustrate prediction quality. This approach confirmed the generalizability and robustness of the model under real-world-like conditions.

Visualizations, including confusion matrices and DTs, were generated using seaborn and scikit-learn's plot_tree, respectively. Where appropriate, pairwise AUROC comparisons between classifiers were evaluated using unpaired two-tailed *t* tests, with statistical significance defined as $p < 0.05$.

Colab Workflow: All machine learning and statistical analyses were conducted using Python (v3.11.5, Python Software Foundation, Wilmington, Delaware, USA) in Google Colab (Google Colaboratory, Google LLC, Mountain View, California, USA). The following libraries were used:

- pandas (v2.2.2, PyData Development Team, NumFOCUS, Austin, Texas, USA) for data wrangling and preparation;
- numpy (v1.26.4, NumPy Developers, NumFOCUS, Austin, Texas, USA) for numerical computations;
- scikit-learn (v1.6.1, Scikit-learn Developers, Inria Foundation, Rocquencourt, France) for RF classification and performance evaluation;
- scipy (v1.14.1, NumFOCUS, Austin, Texas, USA) for chi-square tests and AUROC-based comparisons;
- matplotlib (v3.10.0, Matplotlib Development Team, NumFOCUS, Austin, Texas, USA) and seaborn (v0.13.2, Michael Waskom et al., New York University, New York, USA) for figure generation.[18]

All analyses were performed in a reproducible Google Colab environment, with the full Python script archived for transparency and reusability. The full analysis pipeline and code are available upon request or can be shared as a Google Colab notebook for reproducibility.

The full Python script used for training and visualization of the DT classifier is provided as 'Suppl_Code_File_for_rev15.ipynb'.

Results

Diagnostic performance of individual markers

First, we initially assessed the basic performance of each individual biomarker in distinguishing LC from non-LC cases. To guide the appropriate statistical approach, we tested for a Gaussian distribution each marker using the Shapiro–Wilk test (Table S1). The majority of variables deviated from a Gaussian distribution, supporting the use of non-parametric methods. As such, we applied the Mann–Whitney *U* test to assess group differences and calculated ROC curves to determine diagnostic performance (Table 1).

Among classical serological markers, CA19-9 showed the highest AUROC (0.61), followed by AFP (0.49), CEA (0.42), and bilirubin (0.22). However, all displayed limited standalone accuracy, sensitivity, and specificity. In contrast, several EV-derived markers demonstrated stronger discriminatory potential, with AnnV$^+$EpCAM$^+$CD133$^+$gp38$^+$ large EVs reaching the highest AUROC (0.69), combined with a specificity of 87.4%, and an accuracy of 70.9%. Notably, these EV populations significantly outperformed serological markers, indicating their potential as more specific diagnostic components. Table 1 summarizes the statistical performance of each individual marker.

RF modeling outperforms classical approaches

To identify the most diagnostically relevant marker combinations, we conducted a comparative analysis of multiple logistic regression and RF models, both with and without bilirubin

Table 2. Sorted final model comparison.

EV population	Serological markers	Model	Accuracy	Precision	Recall	F1-score	AUROC
AnnV$^+$EpCAM$^+$CD133$^+$gp38$^+$	AFP, CEA, CA19-9	Random Forest	0.8534	0.8534	0.9019	0.8629	0.9319
AnnV$^+$EpCAM$^+$CD133$^+$gp38$^+$	AFP, CEA, CA19-9	Random Forest	0.8579	0.8584	0.9019	0.8662	0.9317
AnnV$^+$EpCAM$^+$CD133$^+$gp38$^+$	AFP, CEA, CA19-9, Bilirubin	Random Forest	0.8623	0.8584	0.9119	0.8716	0.9315
AnnV$^+$EpCAM$^+$CD133$^+$gp38$^+$	AFP, CEA, CA19-9	Random Forest	0.8624	0.8638	0.9024	0.8704	0.9308
AnnV$^+$EpCAM$^+$CD133$^+$gp38$^+$	AFP, CEA, CA19-9, Bilirubin	Random Forest	0.8491	0.855	0.8924	0.8584	0.9306
AnnV$^+$EpCAM$^+$CD133$^+$gp38$^+$	AFP, CEA, CA19-9	Random Forest	0.8535	0.855	0.9024	0.8638	0.9304
AnnV$^+$EpCAM$^+$CD133$^+$gp38$^+$	AFP, CEA, CA19-9, Bilirubin	Random Forest	0.8713	0.8674	0.9124	0.8788	0.9304
AnnV$^+$EpCAM$^+$CD133$^+$gp38$^+$	AFP, CEA, CA19-9, Bilirubin	Random Forest	0.8536	0.8584	0.8929	0.8617	0.9282
AnnV$^+$EpCAM$^+$CD133$^+$gp38$^+$	AFP, CEA, CA19-9, Bilirubin	Random Forest	0.858	0.8584	0.9024	0.8664	0.9276
AnnV$^+$EpCAM$^+$CD133$^+$gp38$^+$	AFP, CEA, CA19-9	Random Forest	0.8667	0.8584	0.9214	0.877	0.9226
AnnV$^+$EpCAM$^+$CD133$^+$gp38$^+$	AFP, CEA, CA19-9	Logistic Regression	0.6522	0.8571	0.2857	0.4286	0.9029
AnnV$^+$EpCAM$^+$CD133$^+$gp38$^+$	AFP, CEA, CA19-9, Bilirubin	Logistic Regression	0.7174	1.0	0.381	0.5517	0.8305
AnnV$^+$EpCAM$^+$CD133$^+$gp38$^+$	AFP, CEA, CA19-9	Logistic Regression	0.7391	0.8462	0.5238	0.6471	0.8114
AnnV$^+$EpCAM$^+$CD133$^+$gp38$^+$	AFP, CEA, CA19-9, Bilirubin	Logistic Regression	0.7826	1.0	0.5238	0.6875	0.7905
AnnV$^+$EpCAM$^+$CD133$^+$gp38$^+$	AFP, CEA, CA19-9, Bilirubin	Logistic Regression	0.6304	0.7	0.3333	0.4516	0.7657
AnnV$^+$EpCAM$^+$CD133$^+$gp38$^+$	AFP, CEA, CA19-9	Logistic Regression	0.7391	0.9091	0.4762	0.625	0.76
AnnV$^+$EpCAM$^+$CD133$^+$gp38$^+$	AFP, CEA, CA19-9, Bilirubin	Logistic Regression	0.5217	0.3333	0.0476	0.0833	0.7581
AnnV$^+$EpCAM$^+$CD133$^+$gp38$^+$	AFP, CEA, CA19-9	Logistic Regression	0.6739	0.875	0.3333	0.4828	0.7524
AnnV$^+$EpCAM$^+$CD133$^+$gp38$^+$	AFP, CEA, CA19-9	Logistic Regression	0.6522	0.8571	0.2857	0.4286	0.6838
AnnV$^+$EpCAM$^+$CD133$^+$gp38$^+$	AFP, CEA, CA19-9, Bilirubin	Logistic Regression	0.7609	0.9167	0.5238	0.6667	0.6381

Performance metrics (mean ± SD) for the top marker combinations used in the Random Forest model. It includes large extracellular vesicle populations and serological markers, offering insights into how various combinations influence classification metrics. No statistical comparison was performed for this table. AFP, α-fetoprotein; AnnV, Annexin V; CA19-9, carbohydrate antigen 19-9; CEA, carcinoembryonic antigen.

inclusion and without prior feature weighting (N = 166). This model screening, summarized in Table 2, revealed that combinations involving the EV marker AnnV$^+$EpCAM$^+$CD133$^+$gp38$^+$ along with the serological markers AFP, CEA, and CA19-9 consistently ranked among the top-performing configurations across both modeling strategies. Thus, we developed a final RF model incorporating AnnV$^+$EpCAM$^+$CD133$^+$gp38$^+$, AFP, CEA, CA19-9, and bilirubin, and applied a 3 × weighting to the EV marker based on its biological signal strength. This model was evaluated across 10 independent 70/30 train-test splits, yielding

Table 3. Final model Random Forest modeling for liver cancer detection.

Metric	Accuracy	± SD	Precision	± SD	Recall	± SD	F1-score	± SD
70/30	0.882	0.033	0.833	0.055	0.916	0.070	0.870	0.036

Performance of the Random Forest (RF) model over 10 repeated 70/30 train-test splits. Results are expressed as mean ± standard deviation. Performance metrics are derived from 10 repeated 70/30 stratified train-test splits. Bootstrap (n = 1,000) was used to calculate standard deviations.

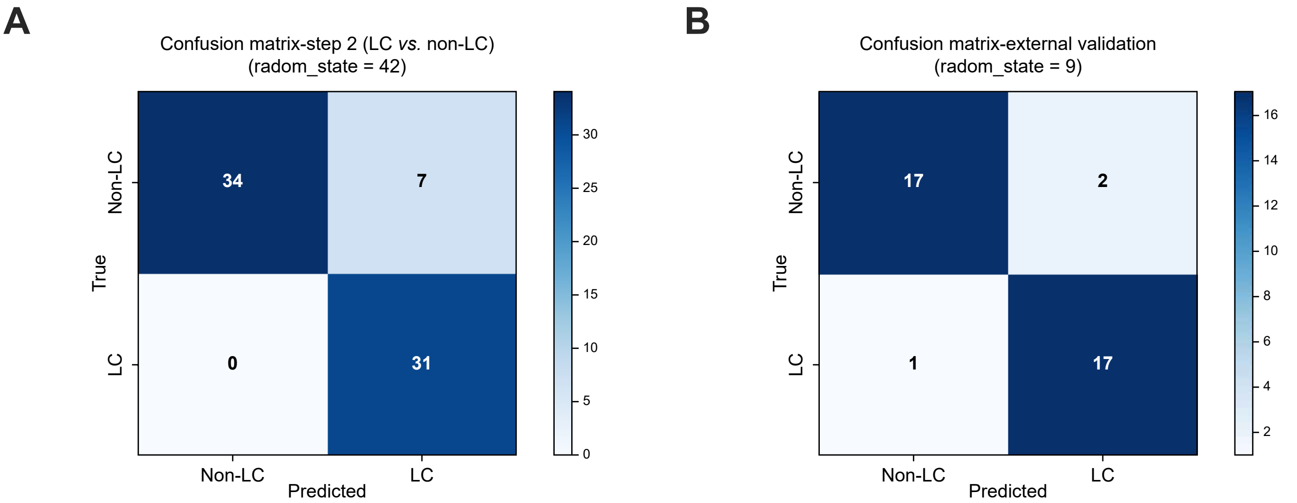

Fig. 1. Confusion matrices for final Random Forest model. (A) Internal validation (Step 2: LC *vs.* Non-LC) using a 70/30 stratified split of the training dataset (random_state = 42). The model was trained using the optimal marker panel consisting of EpCAM/CD133/gp38-positive large extracellular vesicles (EVs) and serological markers AFP, CEA, CA19-9, and bilirubin. The confusion matrix demonstrates a strong classification with 100% sensitivity (no false negatives) and 83% specificity. (B) External validation performance using an independent, previously untrained dataset with complete biomarker profiles (random_state = 9). The same preprocessing pipeline was applied. The model correctly identified 17/18 liver cancer (LC) cases and 17/19 non-LC cases, corresponding to a sensitivity of 94.4% and specificity of 89.5%, demonstrating excellent generalizability across datasets. Color intensity reflects class frequency. AFP, α-fetoprotein; CA19-9, carbohydrate antigen 19-9; CEA, carcinoembryonic antigen; LC, liver cancer; non-LC, non-cancer controls (cirrhosis and healthy).

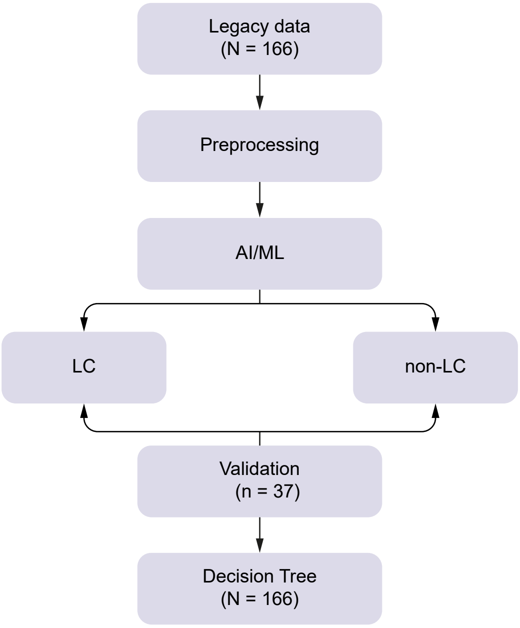

Fig. 2. Schematic overview of the AI-driven re-analysis workflow of legacy large extracellular vesicle (EV) data for liver cancer (LC) detection. The pipeline begins with archived biomarker data ('Legacy Data') followed by pre-processing and feature normalization. Machine learning is performed using a Random Forest classifier, which stratifies cases into LC and non-LC groups. Final model validation is conducted using an external, independently cleaned dataset. A transparent Decision Tree model complements the classification to enhance interpretability.

a mean accuracy of 88.2% ± 3.3%, recall of 91.6% ± 7.0%, precision of 83.3% ± 5.5%, and an F1-score of 87.0% ± 3.6% (Table 3). A representative classification result using a single random split (random_state = 42) is visualized in Fig. 1A, where the model achieved 100% sensitivity (recall) for correctly identifying LC cases and 83% specificity for correctly identifying non-LC samples, yielding an overall accuracy of 90%. These metrics reflect both the clinical utility and generalizability of the final model. Fig. 2 depicts the flowchart for our final RF model for LC screening.

Additionally, we carried out a bootstrap validation (1,000 resamples) of the final RF model. This yielded a mean accuracy of 92.2% ± 4.1%, a precision of 90.4% ± 6.3%, recall of 92.6% ± 6.6%, and F1-score of 91.2% ± 4.6%, with corresponding 95% CIs. These results reinforce the robustness of our RF-based classifier for LC detection and would potentially show any overfitting.

For external validation we used legacy data from our *Liver International* publication, in which large EV isolation/purification and large EV staining and flowcytometric measurements were done by different co-workers to those who had worked on the legacy training data set[10]. Using strict inclusion of only fully complete samples (no imputation), the model achieved a mean accuracy of 86.5% ± 3.5%, precision of 85.0% ± 5.7%, recall of 89.5% ± 4.6%, and F1-score of 87.2% ± 3.7% across 10 stratified runs. The best-performing run (random_state = 9) correctly identified 57 of 59 true LC cases and 43 of 63 non-LC samples, yielding a sensitivity of 96.6% and specificity of 68.3% (Fig. 1B). These

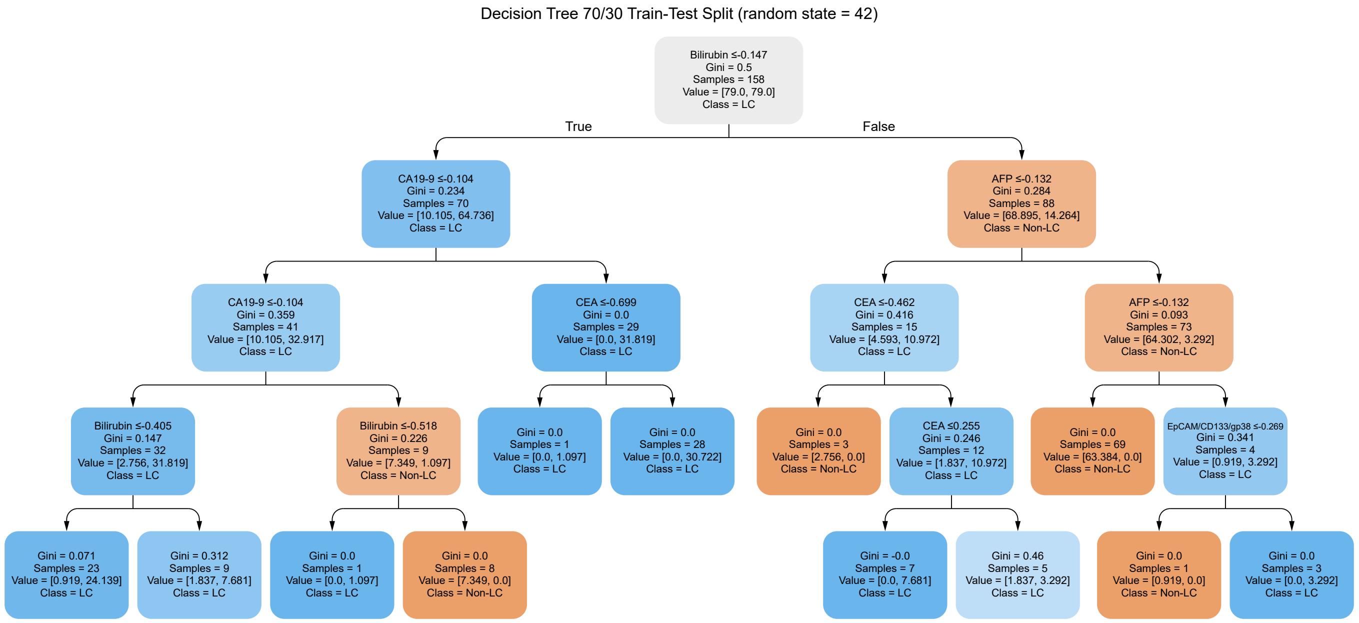

Fig. 3. Decision Tree classifier for liver cancer (LC) detection. This tree illustrates one representative DT model trained on the full dataset (excluding Group 5: Other cancers, N = 166), using five features: AnnV$^+$EpCAM$^+$CD133$^+$gp38$^+$, AFP, CEA, CA19-9, and bilirubin. Each node displays the split criterion, Gini impurity, number of samples, and class distribution (LC *vs.* Non-LC). Blue nodes reflect LC classification, orange nodes indicate Non-LC, and color intensity reflects class purity. While the tree structure may vary across training iterations, this figure demonstrates a valid and interpretable solution. Performance metrics were assessed independently across 10 runs to quantify model robustness (see Fig. 4). For readability, the full EV marker AnnV$^+$EpCAM$^+$CD133$^+$gp38$^+$ is abbreviated as EpCAM$^+$CD133$^+$gp38$^+$ within the Decision Tree nodes. AFP, α-fetoprotein; CA19-9, carbohydrate antigen 19-9; CEA, carcinoembryonic antigen; DT model, Decision Tree model.

results support both the robustness and translational potential of the model in external validation, even under non-ideal conditions.

DT-based modeling for LC detection

In parallel to the RF model, we constructed a standalone DT classifier using the same biomarker panel: AnnV$^+$EpCAM$^+$CD133$^+$gp38$^+$, AFP, CEA, CA19-9, and bilirubin. The goal was to derive an interpretable, transparent decision-support tool that could guide clinical reasoning at the bedside. The final tree structure (Fig. 3) was derived from the full dataset (N = 166, after exclusion of Group 5) using the Gini impurity index to identify optimal binary splits. To rigorously assess model stability and generalizability, we performed 10-fold cross-validation across six distinct training set sizes (50%, 60%, 70%, 80%, 90%, and full fit). The best balance between sensitivity and precision was achieved using a 90/10 training/test ratio, yielding an average accuracy of 0.872 ± 0.066, precision of 0.834 ± 0.071, recall of 0.904 ± 0.074, and F1-score of 0.867 ± 0.067 (Table 4). Performance for the standard 70/30 split remained comparably high, with an average accuracy of 0.866 ± 0.056 and F1-score of 0.858 ± 0.049, confirming the reliability of the model across multiple resampling strategies. As expected, smaller training cohorts (*e.g.* 50/50 split) produced larger performance variability, while the full-fit model (no test set) reflected the maximal achievable structure. The structure of the final representative tree (Fig. 3) revealed bilirubin as the primary discriminator, followed by EV-derived AnnV$^+$EpCAM$^+$CD133$^+$gp38$^+$, and serum markers including AFP and CEA. The DT model trained on the selected five-marker panel (70/30 train-test split, max_depth = 4) achieved a sensitivity of 93.5% and a specificity of 78.4% and an AUROC of 0.849, as illustrated in the confusion matrix (Fig. S1), confirming the strong potential of the model for discriminating LC from non-LC cases.

DT-based clinical thresholds

The final DT model (max_depth = 4) selected all five markers for classification, producing seven decision nodes (Fig. 3). Notably, the EV-based marker AnnV$^+$EpCAM$^+$CD133$^+$gp38$^+$ appeared in two split nodes, highlighting its consistent discriminatory power in LC detection. Thresholds are provided as z-transformed values. Where possible, the thresholds were compared to known clinical reference ranges. For instance, CA19-9 was split at a standardized value below its clinical cutoff of 37 U/ml, and CEA at a z-score corresponding approximately to 5 ng/ml. Although the DT operates on standardized data, this alignment supports its relevance in clinical interpretation. These thresholds, combined with their associated clinical reference values, offer a high degree of transparency and can support future efforts in implementation and prospective validation. For complete details, see Table S2.

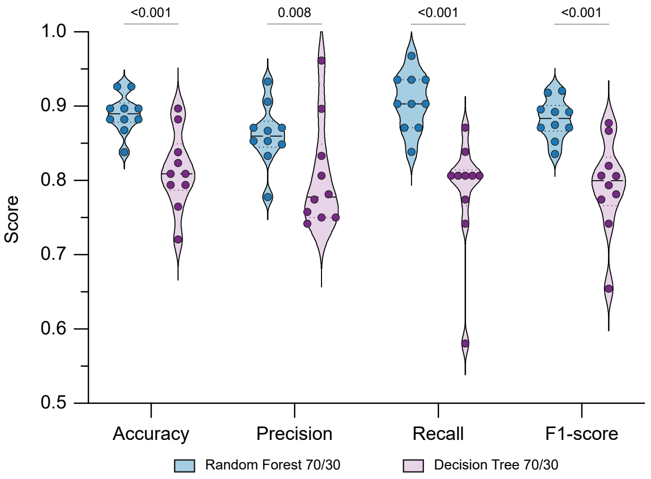

Fig. 4. Comparative diagnostic performance of Random Forest and Decision Tree models for liver cancer (LC) classification. Both models were trained to distinguish LC from non-cancer (non-LC) cases using the same biomarker panel: AnnV$^+$EpCAM$^+$CD133$^+$gp38$^+$ large extracellular vesicles, AFP, CEA, CA19-9, and bilirubin. Each classifier was evaluated across 10 independent 70/30 train-test splits. Shown are individual run values alongside group means for accuracy, precision, recall, and F1-score, with error bars representing standard deviations. Significance was analyzed with GraphPad Prism (Version 10.4.1.) using a non-parametric two-sided Mann–Whitney *U* test (*p* values as depicted). The Random Forest model consistently outperformed the Decision Tree across all metrics, with especially marked improvements in recall and F1-score, highlighting its diagnostic robustness. AFP, α-fetoprotein; AnnV, Annexin V; CA19-9, carbohydrate antigen 19-9; CEA, carcinoembryonic antigen.

To compare the performance and stability of the two classifier types, both the RF and DT models were evaluated across 10 independent 70/30 train-test splits using the same biomarker panel. As shown in Fig. 4, the RF model consistently achieved higher accuracy, recall, and F1-score compared with the DT, with narrower standard deviations indicating greater robustness. Although precision values were comparable between models and did not reach statistical significance, the superior recall and F1-score of the RF model underline its suitability for a screening-oriented diagnostic context.

Supplemental modeling: RF for HCC *vs.* iCCA

To assess whether our model can also distinguish between LC subtypes, we applied the same AI-assisted workflow to the HCC *vs.* iCCA subgroup. Based on individual marker performance, the best discriminative potential was observed

Table 4. Comparative performance of Decision Tree classifiers across multiple training/test split strategies using 10-fold cross-evaluation.

DT split	Accuracy	± SD	Precision	± SD	Recall	± SD	F1-score	± SD
50/50	0.779545	0.156745	0.736429	0.280771	0.726667	0.283549	0.722525	0.265322
60/40	0.859341	0.093832	0.867641	0.137947	0.869048	0.162969	0.846559	0.097923
70/30	0.866250	0.055760	0.859524	0.111986	0.873214	0.076786	0.858073	0.049323
80/20	0.805556	0.103190	0.826587	0.151970	0.741667	0.143829	0.772626	0.125073
90/10	0.872381	0.065804	0.834141	0.070779	0.904444	0.074237	0.867051	0.066901
Full Fit	0.796640	0.087781	0.784719	0.097573	0.776364	0.098434	0.777479	0.088176

Diagnostic performance of Decision Tree models trained on the full dataset (excluding Group 5: Other cancers; N = 166) using six different data partitioning strategies: 50/50, 60/40, 70/30, 80/20, 90/10 train-test splits, and a full-fit model without hold-out data. Each configuration was subjected to 10-fold cross-evaluation. Performance metrics (accuracy, precision, recall, and F1-score) are reported as mean ± standard deviation (SD). These results highlight the influence of training sample size on model robustness and allow transparent comparison across data resampling strategies.

for the EV marker $AnnV^+EpCAM^+CD133^+gp38^+$ in combination with classical serological parameters. The top-ranked marker combinations (Table S4) consistently included AFP and bilirubin. A final RF model using the three-marker panel (AFP, bilirubin, and $Annv^+EpCAM^+CD133^+gp38^+$) achieved a mean accuracy of 66.1% ± 7.0%, a precision of 75.0% ± 8.0%, a recall of 73.0% ± 4.6%, and an F1-score of 73.7% ± 4.5% across 10 stratified 70/30 train-test splits (Table S5). Although the recall for HCC remained high, classification of CCA cases was limited (specificity 45.5%), as visualized in the confusion matrix (Fig. S2). These results support the potential of EV markers in LC sub-classification, albeit with caution because of the known biological overlap between HCC and iCCA.

Discussion

In this study, we revisited a previously published dataset of $AnnV^+$ large EVs originally analyzed for LC detection nearly a decade ago. Now, using modern AI-assisted methods, we demonstrated that legacy data may harbor renewed clinical insight when re-analyzed with more refined, integrative approaches. Hence, we systematically explored existing legacy data of various combinations of large EV-derived and serological markers for LC detection, partly published by us previously,[1,10] or even not published while their individual clinical relevance as measured by AUROC was little. We took advantage of various AI models, both RF and DT classifiers demonstrated strong diagnostic performance, with the final RF model achieving an accuracy of 88.2%, recall of 91.6%, and F1-score of 87.0%. Notably, this surpassed the performance reported in our original 2017 study (AUC 0.70, sensitivity 75%, specificity 47%),[1] and confirms the hypothesis that legacy data might bear novel insight, which was present at that time but obscured by limited analytical power.

One of the most striking findings is the central role as played by a very rare large EV population defined as $AnnV^+EpCAM^+CD133^+gp38^+$, likely reflective of a subtype of immune competent liver progenitor-like cells as published by the lab of Lukacs-Kornek earlier.[19,20] This rare large EV population not only outperformed classical markers like AFP, CEA, and CA19-9 when used alone but also emerged consistently across top-performing marker combinations in both RF and DT models. Although previous studies have focused on exosome-based signatures or bulk plasma markers, our results support a somewhat neglected direction: surface-defined large EV profiling by flow cytometry as a viable and practical clinical tool.[1,10,11,15]

Importantly, classical serological markers alone, although moderately effective in ensemble models, fall short in performance when used in isolation – consistent with prior reports on their limited specificity.[1,10] Importantly, we did not simply add large EV data to an existing framework, but allowed the AI models to test hundreds of combinations and extract data-driven synergy between EV and serological markers. Bilirubin, despite being traditionally viewed as a confounding marker in liver disease, contributed modestly in certain RF model variants, suggesting that even imperfect markers can offer complementary value when integrated with higher-order pattern recognition. From a translational standpoint, the final DT offers clinicians a transparent, interpretable screening tool built upon data-trained logic, without the need for deep machine-learning expertise. Although the RF model is superior in terms of pure performance, the DT model may serve as a low-barrier entry point for implementation or validation studies, especially in lower-resource settings.

To ensure robustness and transparency, we evaluated the DT model across multiple training/testing ratios using 10-fold cross-validation. The model achieved its highest average performance at a 90/10 split (accuracy: 87.2% ± 6.6%; recall: 90.4% ± 7.4%; F1-score: 86.7% ± 6.7%), but also demonstrated strong results at the classical 70/30 ratio (accuracy: 86.6% ± 5.6%; F1-score: 85.8% ± 4.9%). The consistency across different sampling strategies reflects the model's reliability despite the moderate dataset size. Moreover, in the representative 70/30 configuration, we observed a sensitivity of 93.5% and a specificity of 78.4%, suggesting clinically meaningful diagnostic behavior, especially when early detection is prioritized over false positives. These results contrast sharply with our original 2017 results (sensitivity: 75%, specificity: 47%) and underline the added value of AI in optimizing marker synergy from even modest datasets.[1]

Of note, we should not step away from exploring novel markers that may be used as a sufficient single marker, including EVs and their subpopulations. Others achieved the same or better AUROC values as we did here. Recent studies have highlighted the diagnostic potential of EV-associated biomarkers in LC. A phase II study picked up our earlier finding on CD147 and EpCAM positive EVs and developed extensively further towards an AI-modeled HCC EV ECG Score consisting of small EV subpopulations ($EpCAM^+$ $CD63^+$, $CD147^+$ $CD63^+$, and $GPC3^+$ $CD63^+$ HCC EVs) for detecting early-stage HCC. Sun *et al.*[21] reported an overall AUROC of 0.95 with a sensitivity of 91% and a specificity of 90%. To use AI-driven models including bootstrapping for analysis multifactorial EV data as done by von Felden *et al.*[22] is not common, but enhances the chance to detect non-obvious dependencies and correlations among markers. Here, von Felden *et al.*[22] developed an exRNA-derived smRC HCC-specific signature with an associated sensitivity of 86% and a specificity of 91%. It is evident that a future liquid biopsy marker will be likely the result of a multifactorial approach with various markers of the same type or different types as EVs and classical serological markers. Our own publication in 2020 also showed a synergistic effect if $AnnV^+CD44v6^+$ large EVs and AFP had been combined to differentiate HCC from CCA.[10] Our current AI-assisted approach is not aimed to surpass and be superior to others, but rather to show that legacy data might be an overlooked source of novel insights.

We performed an additional subgroup analysis to explore whether our model could distinguish between HCC and iCCA. Although we observed modest improvements compared with the original 2017 publication, a clear and clinically actionable separation remains challenging. This aligns with the broader consensus that HCC and CCA share overlapping biological signatures, especially in livers of patients with cirrhosis, and may not be reliably distinguished using serological and EV markers alone. Notably, our AI-derived model achieved high sensitivity for HCC, but specificity for iCCA remained limited – highlighting the need for complementary imaging (*e.g.* Liver Imaging Reporting and Data System [LI-RADS]) and future integration of more specific biomarkers such as ctDNA,

methylation patterns, or transcript fusion profiles. Moreover, we acknowledge that established composite biomarker scores such as GALAD, incorporates AFP, AFP-L3%, and DCP, as well as gender and age. were not assessable in this legacy dataset.[23] Future studies integrating GALAD or related indices may help further improve predictive performance and risk stratification. Nevertheless, these results support the potential of EV markers as part of a broader diagnostic framework but emphasize that their role in HCC *vs.* CCA discrimination is still exploratory and hypothesis-generating.

Certainly, our study is not without limitations. Our model remains internally validated and externally with a small validation cohort (n = 37); future work must apply it to external multicentric patient cohorts, hence, ideally across different populations and clinical contexts. Furthermore, the presence of imputed values for serological markers – a necessity owing to the legacy dataset structure – underscores the need for prospective, multicenter validation with full datasets. Of note, it remains debatable whether $AnnV^+$ large EVs, which cannot maintain plasma membrane asymmetry because of the absence of ongoing metabolism, originate from viable cells or rather reflect apoptotic cell remnants. However, in the context of marker exploration, this distinction is of secondary importance. It is also important to note that lipoproteins may expose phosphatidylserine to some degree and could therefore serve as potential binding partners for AnnV, possibly contributing to false-positive EV signals, as discussed by Botha *et al.*[24] Nonetheless, lipoproteins typically fall within the size range of small EVs (30–180 nm) and do not express transmembrane markers such as EpCAM, ASGPR1, or gp38. Moreover, membrane-bound proteins cannot be embedded in the lipid monolayer surface of lipoproteins.[24] We acknowledge that inflammatory or fibrotic processes – particularly in patients with cirrhosis – can obscure tumor-specific biomarkers, potentially reducing diagnostic specificity. This biological overlap has historically complicated efforts to develop reliable, non-invasive markers for LC. In addition, ultrasound-based detection remains highly operator-dependent. We previously discussed this challenge in detail in the context of large EVs and ultrasound staging in liver disease.[11] These limitations further highlight the need for robust, objective, and reproducible strategies such as the AI-assisted, multimodal approach proposed in our present study.

Despite these limitations, our work highlights a much broader paradigm: data do not expire, only the tools to unlock its meaning do. Here, by applying AI-assisted, clinically informed modeling, we were able to resurrect hidden insights and build towards a potentially deployable screening framework for LC based on large EVs – a biological signal largely overlooked until now. Although AI/machine learning has only rarely been applied to biomarker discovery in clinical oncology, previous work by Kaluri *et al.*[25] demonstrated its feasibility. To our knowledge, the present study is the first to apply supervised AI models to legacy EV data in LC, enabling improved screening-oriented classification through integration of serological and EV-based features.

As AI continues to permeate modern healthcare, its impact extends well beyond diagnostics. Surgical systems such as the da Vinci® Robotic Platform – already used in hepatobiliary and oncologic procedures – demonstrate how AI and robotics can enhance intraoperative precision, optimize motion control, and support clinical decision-making in real time.[26,27] These platforms incorporate AI-driven features including tremor suppression, motion scaling, and even early-stage image-guided augmentation. In this broader context, our AI-assisted biomarker model reflects a parallel evolution on the diagnostic front – automating pattern recognition and risk assessment for LC using legacy data. Taken together, these developments suggest an emerging synergy between AI-based early detection and AI-augmented intervention. This convergence highlights a future where LC care may be driven by a continuous AI-enhanced workflow – from risk stratification and screening to resection and postoperative follow-up.

Beyond its immediate relevance for LC screening, this AI-assisted re-analysis may serve as a blueprint for the retrospective evaluation of legacy datasets across various clinical domains, including chronic liver diseases[11] and multifactorial conditions such as polytrauma,[16,17] where complex biomarker patterns often remain underexplored.

In summary, our study illustrates how the re-exploitation of legacy data using modern AI tools can yield in novel clinically relevant diagnostic insights. By integrating rare, surface-defined large EVs with classical serological markers through machine-learning models, we were able to exceed the diagnostic performance of each marker if used single, including our own earlier efforts. Notably, we present two complementary tools – a high-performance RF classifier and a transparent DT model – that may serve different clinical needs and infrastructure settings. This work not only promotes large EVs as promising cancer liquid biopsy markers, but also advocates for a broader rethinking of data reuse in biomedical research. When analyzed thoughtfully, even 'old' data can reveal new answers. Viewed in the broader clinical context, this evolution mirrors the integration of AI in surgical technologies such as the da Vinci platform – collectively reflecting a future in which AI informs both diagnosis and intervention across the cancer care continuum.

Affiliations
[1]*Department of General, Visceral and Thoracic Surgery, German Armed Forces Central Hospital, Koblenz, Germany;* [2]*Department of Medicine II, Saarland University Medical Center, Saarland University, Homburg, Germany;* [3]*Department of Gastroenterology, Hepatology and Transplant Medicine, Medical Faculty, University of Duisburg-Essen, Essen, Germany;* [4]*Laboratory of Metabolic Liver Diseases, Department of General, Transplant and Liver Surgery, Centre for Preclinical Research, Medical University of Warsaw, Warsaw, Poland;* [5]*Department of Liver and Gastrointestinal Diseases, Biogipuzkoa Health Research Institute, Donostia University Hospital, University of the Basque Country (UPV/EHU), Ciberhed, Ikerbasque, San Sebastian, Spain;* [6]*Department of Biochemistry and Genetics, School of Sciences, University of Navarra, Pamplona, Spain;* [7]*Babeş-Bolyai University - UBB Med Department, Regional Institute of Gastroenterology and Hepatology, Cluj-Napoca, Romania;* [8]*Department of Integrated Oncology, Center for Integrated Oncology (CIO), University Hospital of Bonn, 53127, Bonn, Germany;* [9]*Institute of Molecular Medicine and Experimental Immunology, University Hospital of the Rheinische Friedrich-Wilhelms-University, 53127 Bonn, Germany*

Abbreviations

AFP, α-fetoprotein; AFP-L3, lectin-bound AFP; AI, artificial intelligence; AASLD, American Association for the Study of Liver Diseases; ALT, alanine transaminase; AnnV, Annexin V; ASGPR1, asialoglycoprotein receptor 1; AST, aspartate transaminase; AUROC, area under the ROC curve; CA19-9, carbohydrate antigen 19-9; CCA, cholangiocarcinoma; CEA, carcinoembryonic antigen; CD133, cluster of differentiation 133; CI, confidence interval; DCP, des-gamma carboxy-prothrombin; DT model, Decision Tree model; EpCAM, epithelial cell adhesion molecule; EVs, extracellular vesicles; FDA, Food and Drug Administration; GGT, gamma-glutamyl transferase; gp38, glycoprotein 38; HCC, hepatocellular carcinoma; iCCA, intrahepatic cholangiocellular carcinoma; ISEV, International Society for Extracellular Vesicles; LASSO, least absolute shrinkage and selection operator; LC, liver cancer; LI-RADS, Liver Imaging Reporting and Data System; MISEV, Minimal Information for Studies of Extracellular Vesicles; PCA, principal component analysis; PS, phosphatidylserine; RF model, Random Forest model; ROC, receiver operating characteristic; SD, standard deviation.

Financial support

Studies were supported by the Deutsche Forschungsgemeinschaft (DFG, German Research Foundation) to MTK (DFG project number 410853455). VL-K is funded by the Deutsche Forschungsgemeinschaft (DFG, German Research Foundation) under Germany‘s Excellence Strategy – EXC 2151 – 390873048 and DFG Project number 411345524 and 432325352.

Conflicts of interest

The authors have no conflicts of interest to declare.

Please refer to the accompanying ICMJE disclosure forms for further details.

Authors' contributions

EV methodology: HJ-H, VL-K, MTK. Provided human samples and human data management: AGW, MK, JMB, TM, AK, CG, SS, RS, IGHSW. Experimental EV data management & analysis: HJ-H, SKG, MTK. Experimental data interpretation: AGW, VL-K, MTK. Funding acquisition: AGW, RS, MTK. Writing – original draft: AGW, VL-K, MTK. Writing – review and editing: all authors. Original study design: VL-K, MTK. Project supervision: MTK.

Data availability

The datasets analyzed during the current study are derived from a legacy EV dataset previously published by our group (*Journal of Hepatology*, 2017) and have been reprocessed for this analysis. All Python code used for RF and DT model training, validation, and visualization – as well as example input files – will be made available to qualified researchers upon reasonable request to the corresponding author.

The decision to share these materials will follow ethical data handling guidelines and institutional approval requirements. All shared materials will include well-documented Python notebooks (Google Colab compatible) for reproducibility.

Acknowledgements

This article is based upon work from the European Network for the Study of Cholangiocarcinoma and the COST Action CA22125 Precision medicine in biliary tract cancer (Precision-BTC-Network) supported by COST (European Cooperation in Science and Technology: www.cost.eu) to MK, JMB and VL-K.

Declaration of generative AI and AI-assisted technologies in the writing process

We acknowledge the use of OpenAI's ChatGPT and Google's Colab for support in Python coding, data analysis refinement, to improve language and readability, and methodological clarity throughout the re-evaluation process. After using this tool/service, the author(s) reviewed and edited the content as needed and take(s) full responsibility for the content of the publication.

Supplementary data

Supplementary data to this article can be found online at https://doi.org/10.1016/j.jhepr.2025.101540.

References

Author names in bold designate shared co-first authorship.

[1] **Julich-Haertel H**, **Urban SK**, **Krawczyk M**, et al. Cancer-associated circulating large extracellular vesicles in cholangiocarcinoma and hepatocellular carcinoma. J Hepatol 2017;67:282–292.
[2] Beam AL, Kohane IS. Big data and machine learning in health care. JAMA 2018;319:1317–1318.
[3] European Association for the Study of the Liver. EASL Clinical Practice Guidelines on the management of hepatocellular carcinoma. J Hepatol 2025;82:315–374.
[4] Singal AG, Llovet JM, Yarchoan M, et al. AASLD Practice Guidance on prevention, diagnosis, and treatment of hepatocellular carcinoma. Hepatology 2023;78:1922–1965.
[5] Tsai SL, Huang GT, Yang PM, et al. Plasma des-gamma-carboxyprothrombin in the early stage of hepatocellular carcinoma. Hepatology 1990;11:481–488.
[6] Zhou JM, Wang T, Zhang KH. AFP-L3 for the diagnosis of early hepatocellular carcinoma: a meta-analysis. Medicine (Baltimore) 2021;100:e27673.
[7] Gines P, Krag A, Abraldes JG, et al. Liver cirrhosis. Lancet 2021;398:1359–1376.
[8] Banales JM, Feldstein AE, Sanger H, et al. Extracellular vesicles in liver diseases: meeting report from the international liver congress 2018. Hepatol Commun 2019;3:305–315.
[9] **Urban SK**, **Mocan T**, Sanger H, et al. Extracellular vesicles in liver diseases: diagnostic, prognostic, and therapeutic application. Semin Liver Dis 2019;39:70–77.
[10] **Urban SK**, **Sanger H**, **Krawczyk M**, et al. Synergistic effects of extracellular vesicle phenotyping and AFP in hepatobiliary cancer differentiation. Liver Int 2020;40:3103–3116.
[11] Kornek M, Lynch M, Mehta SH, et al. Circulating microparticles as disease-specific biomarkers of severity of inflammation in patients with hepatitis C or nonalcoholic steatohepatitis. Gastroenterology 2012;143:448–458.
[12] **Yanez-Mo M**, **Siljander PR**, Andreu Z, et al. Biological properties of extracellular vesicles and their physiological functions. J Extracell Vesicles 2015;4:27066.
[13] van Niel G, D'Angelo G, Raposo G. Shedding light on the cell biology of extracellular vesicles. Nat Rev Mol Cell Biol 2018;19:213–228.
[14] Lukacs-Kornek V, Julich-Haertel H, Urban SK, et al. Multi-surface antigen staining of larger extracellular vesicles. Methods Mol Biol 2017;1660:201–208.
[15] Willms A, Muller C, Julich H, et al. Tumour-associated circulating microparticles: a novel liquid biopsy tool for screening and therapy monitoring of colorectal carcinoma and other epithelial neoplasia. Oncotarget 2016;7:30867–30875.
[16] Wohler A, Gries SK, Salzmann RJS, et al. Monocyte derived large extracellular vesicles in polytrauma. J Extracell Biol 2024;3:e70005.
[17] Wang B, Wohler A, Greven J, et al. Liquid biopsy in organ damage: small extracellular vesicle chip-based assessment of polytrauma. Front Immunol 2023;14:1279496.
[18] Waskom ML. seaborn: statistical data visualization. J Open Source Software 2021;6:3021.
[19] **Eckert C**, **Kim YO**, **Julich H**, et al. Podoplanin discriminates distinct stromal cell populations and a novel progenitor subset in the liver. Am J Physiol Gastrointest Liver Physiol 2016;310:G1–G12.
[20] Lukacs-Kornek V, Lammert F. The progenitor cell dilemma: cellular and functional heterogeneity in assistance or escalation of liver injury. J Hepatol 2017;66:619–630.
[21] **Sun N**, **Zhang C**, **Lee YT**, et al. HCC EV ECG score: an extracellular vesicle-based protein assay for detection of early-stage hepatocellular carcinoma. Hepatology 2023;77:774–788.
[22] von Felden J, Garcia-Lezana T, Dogra N, et al. Unannotated small RNA clusters associated with circulating extracellular vesicles detect early stage liver cancer. Gut 2021 Jul 28. https://doi.org/10.1136/gutjnl-2021-325036. gutjnl-2021-325036.
[23] Best J, Bilgi H, Heider D, et al. The GALAD scoring algorithm based on AFP, AFP-L3, and DCP significantly improves detection of BCLC early stage hepatocellular carcinoma. Z Gastroenterol 2016;54:1296–1305.
[24] Botha J, Handberg A, Simonsen JB. Lipid-based strategies used to identify extracellular vesicles in flow cytometry can be confounded by lipoproteins:

evaluations of annexin V, lactadherin, and detergent lysis. J Extracell Vesicles 2022;11:e12200.
[25] Li B, Kugeratski FG, Kalluri R. A novel machine learning algorithm selects proteome signature to specifically identify cancer exosomes. eLife 2024;12.
[26] Minamimura K, Aoki Y, Kaneya Y, et al. Current status of robotic hepatobiliary and pancreatic surgery. J Nippon Med Sch 2024;91:10–19.
[27] Spiegelberg J, Iken T, Diener MK, et al. Robotic-assisted surgery for primary hepatobiliary tumors-possibilities and limitations. Cancers (Basel) 2022;14:265.

Keywords: Artificial intelligence; Random forest; Liver cancer; Cancer screening; Cirrhosis; Large extracellular vesicles; Ectosomes; Biomarker; Liquid biopsy; Additive scoring.
Received 11 April 2025; received in revised form 23 July 2025; accepted 24 July 2025; Available online 6 August 2025

Journal of Hepatology, Volume *7*

Supplemental information

What we missed then, AI sees now: Revisiting legacy large extracellular vesicle data to reveal synergistic biomarkers for liver cancer screening

Arnulf G. Willms, Marcin Krawczyk, Henrike Julich-Haertel, Sabine K. Gries, Jesus M. Banales, Tudor Mocan, Angelina Klein, Sebastian Schaaf, Christoph Güsgen, Robert Schwab, Ingo G.H. Schmidt-Wolf, Veronika Lukacs-Kornek, and Miroslaw T. Kornek

What we missed then, AI sees now: Revisiting legacy large extracellular vesicle data to reveal synergistic biomarkers for liver cancer screening

Arnulf G. Willms, Marcin Krawczyk, Henrike Julich-Haertel, Sabine K. Gries, Jesus M. Banales, Tudor Mocan, Angelina Klein, Sebastian Schaaf, Christoph Güsgen, Robert Schwab, Ingo G.H. Schmidt-Wolf, Veronika Lukacs-Kornek, Miroslaw T. Kornek

Table of contents

Supplementary Materials and Methods……………………………………………..……2

Fig. S1……………………………………………………………………………….....4

Fig. S2………………………………………………………………………………… 4

Table S1…………………………………………………………………………………5

Table S2…………………………………………………………………………………5

Table S3…………………………………………………………………………………5

Table S4…………………………………………………………………………………6

Table S5…………………………………………………………………………………7

Python Codes for LC vs. Non-LC……………………………………………...……...8

Supplementary Materials and Methods – HCC vs. CCA Classification

Dataset and Cohort Composition

This analysis was conducted on a previously published and annotated legacy dataset used in our earlier liver cancer biomarker study. For the present HCC vs. CCA re-analysis, only individuals labeled as "Group1: HCC" and "Group2: CCA" were included. The dataset contained flow cytometric measurements of large extracellular vesicle (EV) populations and classical serological markers, collected under ethically approved study protocols as described in the main manuscript. All samples were de-identified prior to analysis.

Feature Selection and Marker Panel Definition

The feature pool was identical to the LC vs. non-LC workflow and included both large EV-based markers and classical serological parameters. Initially, exploratory univariate ROC analyses were performed for each marker and selected combinations to assess individual diagnostic potential. The EV marker **AnnV$^+$EpCAM$^+$CD133$^+$gp38$^+$** showed the strongest discriminative power between HCC and iCCA cases. This EV marker was subsequently combined with serological parameters — specifically **AFP**, **Bilirubin**, and **CEA** — in various configurations to identify the most diagnostically robust panel.

Machine Learning Pipeline and Model Training

The final marker panel included the EV marker **AnnV$^+$EpCAM$^+$CD133$^+$gp38$^+$**, along with **AFP** and **Bilirubin**. This feature set was used to train and evaluate a **Random Forest (RF)** classifier. Input data was standardized using z-score normalization, and the EV marker was weighted 3× to emphasize its biological importance. The model was implemented in Python using scikit-learn, with the following pipeline:

- Data split: Stratified 70/30 train-test split
- Classifier: RandomForestClassifier(n_estimators=200,
  class_weight='balanced')
- Repeated evaluation: 10 runs with different random_state values (0–9)
- Metrics: Accuracy, precision, recall (sensitivity), F1-score, and AUROC

Statistical Analysis

No additional imputation was necessary, as only complete cases for the selected markers were included. Marker distributions were assessed using the Shapiro–Wilk test, and comparisons between HCC and iCCA were performed using **Mann–**

Whitney U tests. Model performance variability was quantified using standard deviation across 10 random stratified runs.

Supplementary Results

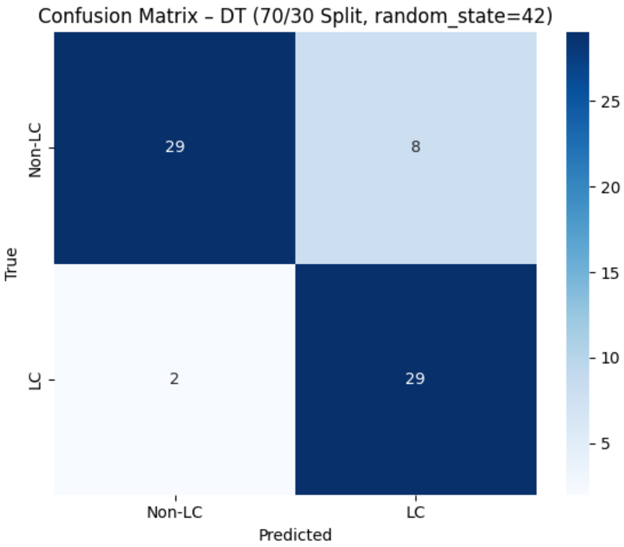

Fig. S1. Confusion matrix of the Decision Tree model (max_depth = 4, 70/30 stratified train-test split, random_state = 42) applied to the five-marker panel distinguishing between LC and non-LC patients. The matrix shows the counts of true positives (LC correctly predicted), true negatives (non-LC correctly predicted), false positives, and false negatives. The classifier achieved a sensitivity of 93.5% and a specificity of 78.4%.

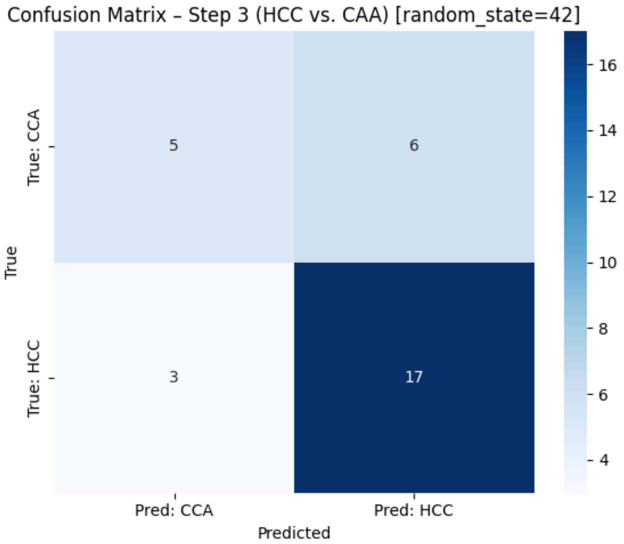

Fig. S2. Confusion matrix of the Random Forest classifier distinguishing HCC from CCA using the marker combination AFP + Bilirubin + AnnV$^+$EpCAM$^+$CD133$^+$gp38$^+$. Results are based on a stratified 70/30 train-test split (random_state=42). The model correctly classified 17 of 20 HCC cases (sensitivity: 85.0%) and 5 of 11 CCA cases (specificity: 45.5%), with an overall accuracy of 71.0%. Color intensity reflects class frequency.

Table S1. Shapiro-Wilk Normality Test for Marker Distributions

Marker	LC p-value	Non-LC p-value	LC Normal?	Non-LC Normal?
**AnnV$^+$EpCAM$^+$**	2.0422498414607304e-10	4.169339717918774e-08	No	No
**AnnV$^+$EpCAM$^+$ASGPR1$^+$**	3.0680805118699084e-12	1.4123037184177176e-09	No	No
**AnnV$^+$EpCAM$^+$CD133$^+$**	3.7788443706630876e-14	3.9833296649541804e-16	No	No
**AnnV$^+$EpCAM$^+$ASGPR1$^+$CD133$^+$**	1.107709487441404e-17	3.6967995396645924e-13	No	No
**AnnV$^+$EpCAM$^+$CD56$^+$**	4.645055007301835e-09	3.710650710309071e-12	No	No
**AnnV$^+$EpCAM$^+$ASGPR1$^+$gp38$^+$**	3.026721392873725e-20	4.394866429185583e-18	No	No
**AnnV$^+$EpCAM$^+$ASGPR1$^+$gp38$^+$CD133$^+$**	6.876331106403152e-20	2.166946180647443e-19	No	No
**AnnV$^+$EpCAM$^+$gp38$^+$**	1.502857719242987e-18	4.953815281990632e-17	No	No
**AnnV$^+$EpCAM$^+$CD133$^+$gp38$^+$**	2.0880691842653186e-18	2.3345958901760285e-18	No	No
**AnnV$^+$EpCAM$^-$CD56$^+$**	0.0004233425715938	1.1854772719033235e-10	No	No
**AnnV$^+$EpCAM$^-$CD133$^+$**	5.2130597243832224e-18	9.389114885329702e-22	No	No
**AnnV$^+$EpCAM$^-$gp38$^+$**	4.046269612770215e-15	1.8682458358121323e-10	No	No
**AnnV$^+$EpCAM$^-$CD133$^+$gp38$^+$**	1.3912658895775162e-21	1.0446741395157109e-23	No	No
**AFP**	3.972371313588609e-21	2.026842205902332e-19	No	No
**CEA**	5.455321652491732e-17	5.760598230683804e-20	No	No
**CA19-9**	8.339104922205512e-22	4.505197847239439e-19	No	No
**Bilirubin**	1.396737742385436e-16	7.101633133277574e-21	No	No

Table S2. Node-specific marker thresholds and classification decisions derived from the decision tree classifier (max_depth=4, random_state=42) trained on the 70/30 train-test split. This table lists all decision nodes used by the classifier to separate liver cancer (LC) from non-LC cases based on five biomarkers. For each node, the corresponding marker, raw threshold, classification direction, predicted class, Gini impurity, number of samples, and the LC/Non-LC sample distribution are reported. Thresholds refer to z-score standardized input values; the EV marker (AnnV$^+$EpCAM$^+$CD133$^+$gp38$^+$) was weighted ×3 prior to model fitting. All EV marker values are expressed in arbitrary units (a.u.), defined as relative marker signal per 1,000 AnnV$^+$ EVs, to allow intra-assay comparability.

Marker	Node	Z-Score Cut-off	Clinical Cut-off	Unit
**AFP**	N1	-0.132	3.19188	ng/mL
**AnnV$^+$EpCAM$^+$CD133$^+$gp38$^+$**	N13	-0.235	8.4496	a.u.
**CEA**	N2	0.209	4.69712	ng/mL
**CA19-9**	N3	-0.085	102.34015	U/mL
**Bilirubin**	N4	-0.253	1.86266	mg/dL
**AFP**	N5	0.615	6.85965	ng/mL
**AnnV$^+$EpCAM$^+$CD133$^+$gp38$^+$**	N6	-0.235	8.4496	a.u.

Table S3. Diagnostic performance of individual markers in HCC vs. CCA. This table summarizes the diagnostic performance of individual markers, including extracellular vesicle (EV) populations and classical serological parameters, for distinguishing HCC from CCA. Performance metrics include AUROC, optimal thresholds based on the Youden index, sensitivity, specificity, and overall accuracy.

Marker	Best Threshold	Sensitivity	Specificity	Accuracy	F1 Score	MW p-value
**AFP**	5.39	0.701	0.861	0.757	0.79	<0.001
**CA19-9**	18.06	0.388	0.278	0.35	0.437	<0.001
**AnnV$^+$EpCAM$^+$CD133$^+$gp38$^+$**	11.597	0.418	0.333	0.388	0.471	0.013
**Bilirubin**	0.92	0.448	0.389	0.427	0.504	0.017
**AnnV$^+$EpCAM$^-$CD133$^+$gp38$^+$**	0.0	1.0	0.0	0.65	0.788	0.024

**AnnV^{+}EpCAM^{-}CD133^{+}**	3.912	0.463	0.417	0.447	0.521	0.036
**AnnV^{+}EpCAM^{+}ASGPR1^{+}gp38^{+}CD133^{+}**	6.509	0.433	0.361	0.408	0.487	0.055
**AnnV^{+}**	35281.844	0.478	0.444	0.466	0.538	0.138
**AnnV^{+}EpCAM^{+}ASGPR1^{+}**	12.797	0.552	0.583	0.563	0.622	0.145
**AnnV^{+}EpCAM^{+}CD133^{+}**	11.542	0.493	0.472	0.485	0.555	0.151
**AnnV^{+}EpCAM^{-}CD56^{+}**	231.454	0.493	0.472	0.485	0.555	0.159
**AnnV^{+}EpCAM^{+}CD56^{+}**	13.211	0.552	0.583	0.563	0.622	0.163
**AnnV^{+}EpCAM^{+}ASGPR1^{+}CD133^{+}**	27.668	0.478	0.444	0.466	0.538	0.356
**AnnV^{+}EpCAM^{+}ASGPR1^{+}gp38^{+}**	0.0	1.0	0.0	0.65	0.788	0.502
**AnnV^{+}EpCAM^{-}gp38^{+}**	7.273	0.522	0.528	0.524	0.588	0.511
**CEA**	2.44	0.567	0.472	0.534	0.613	0.531
**AnnV^{+}EpCAM^{+}gp38^{+}**	0.0	1.0	0.0	0.65	0.788	0.641
**ALT**	42.0	0.537	0.444	0.505	0.585	0.727
**AnnV^{+}EpCAM^{+}**	96.439	0.522	0.528	0.524	0.588	0.865

Table S4. Sorted Final Model Comparison Table for HCC vs. CCA. This table summarizes the performance metrics for the top marker combinations used in the Random Forest model. It includes large EV populations and serological markers, offering insights into how various combinations influence classification metrics.

EV Marker	Serological Markers	Model	Accuracy	Precision	Recall	F1-Score	AUROC
**AnnV^{+}EpCAM^{+}CD133^{+}gp38^{+}**	AFP, Bilirubin	Random Forest	0.71	0.739	0.85	0.791	0.841
**AnnV^{+}EpCAM^{+}CD133^{+}gp38^{+}**	AFP	Random Forest	0.806	0.818	0.9	0.857	0.832
**AnnV^{+}EpCAM^{+}CD133^{+}gp38^{+}**	AFP, Bilirubin, CEA	Random Forest	0.742	0.773	0.85	0.81	0.827
**AnnV^{+}EpCAM^{+}CD133^{+}gp38^{+}**	AFP, CA19-9, Bilirubin, CEA, ALT	Random Forest	0.71	0.72	0.9	0.8	0.827
**AnnV^{+}EpCAM^{+}CD133^{+}gp38^{+}**	AFP, Bilirubin, ALT	Random Forest	0.645	0.696	0.8	0.744	0.823
**AnnV^{+}EpCAM^{+}CD133^{+}gp38^{+}**	AFP, Bilirubin, CEA, ALT	Random Forest	0.71	0.739	0.85	0.791	0.814
**AnnV^{+}EpCAM^{+}CD133^{+}gp38^{+}**	AFP, CEA	Random Forest	0.677	0.727	0.8	0.762	0.807
**AnnV^{+}EpCAM^{+}CD133^{+}gp38^{+}**	AFP, CA19-9, Bilirubin, ALT	Random Forest	0.71	0.72	0.9	0.8	0.805
**AnnV^{+}EpCAM^{+}CD133^{+}gp38^{+}**	CA19-9, Bilirubin, CEA, ALT	Random Forest	0.677	0.727	0.8	0.762	0.805
**AnnV^{+}EpCAM^{+}CD133^{+}gp38^{+}**	AFP, CA19-9, CEA	Random Forest	0.71	0.739	0.85	0.791	0.802
**AnnV^{+}EpCAM^{+}CD133^{+}gp38^{+}**	AFP, CA19-9, Bilirubin, CEA	Random Forest	0.677	0.708	0.85	0.773	0.8
**AnnV^{+}EpCAM^{+}CD133^{+}gp38^{+}**	AFP, CA19-9, Bilirubin	Random Forest	0.677	0.708	0.85	0.773	0.795

**AnnV⁺EpCAM⁺CD133⁺gp38⁺**	AFP, CA19-9, ALT	Random Forest	0.742	0.731	0.95	0.826	0.791
**AnnV⁺EpCAM⁺CD133⁺gp38⁺**	AFP, CA19-9	Random Forest	0.774	0.76	0.95	0.844	0.78
**AnnV⁺EpCAM⁺CD133⁺gp38⁺**	CA19-9, Bilirubin, CEA	Random Forest	0.677	0.727	0.8	0.762	0.777
**AnnV⁺EpCAM⁺CD133⁺gp38⁺**	AFP, CA19-9, CEA, ALT	Random Forest	0.677	0.708	0.85	0.773	0.768
**AnnV⁺EpCAM⁺CD133⁺gp38⁺**	AFP, CEA, ALT	Random Forest	0.742	0.8	0.8	0.8	0.768
**AnnV⁺EpCAM⁺CD133⁺gp38⁺**	CA19-9, Bilirubin, ALT	Random Forest	0.677	0.727	0.8	0.762	0.761
**AnnV⁺EpCAM⁺CD133⁺gp38⁺**	AFP, ALT	Random Forest	0.645	0.696	0.8	0.744	0.759
**AnnV⁺EpCAM⁺CD133⁺gp38⁺**	CA19-9, CEA, ALT	Random Forest	0.645	0.696	0.8	0.744	0.748

Table S5. Final Model Random Forest Modeling for HCC vs. CCA Detection Performance of the Random Forest (RF) model over a 70/30 train-test split for distinguishing HCC from CCA using the marker combination AFP + Bilirubin + AnnV⁺EpCAM⁺CD133⁺gp38⁺. Results are derived from a single run with random_state=42.

**Metric**	**Accuracy**	**±SD**	**Precision**	**±SD**	**Recall**	**±SD**	**F1-Score**	**±SD**
**70/30**	0.61	0.070	0.739	0.080	0.850	0.046	0.791	0.045

Python Codes for LC vs. Non-LC

Python Random Forest Code LC vs. Non-LC

```
# === IMPORT LIBRARIES ===
import pandas as pd
import numpy as np
from sklearn.ensemble import RandomForestClassifier
from sklearn.model_selection import train_test_split
from sklearn.metrics import (
    accuracy_score, precision_score, recall_score, f1_score,
    classification_report, confusion_matrix
)
```

```
from sklearn.preprocessing import StandardScaler
import seaborn as sns
import matplotlib.pyplot as plt

# === 1. LOAD AND PREPARE DATA ===
df = pd.read_csv("Data_4_Group4_Filled_From_Group3_FIXED_Final.csv")
print("Sample Distribution:", df["LC_label"].value_counts().to_dict())

# Define feature groups
ev_markers = ["EpCAM/CD133/gp38"]
serological_markers = ["AFP", "CEA", "CA19-9", "Bilirubin"]
all_markers = ev_markers + serological_markers

df["LC_label"] = df["LC_label"].astype(int)
X = df[all_markers].copy()
y = df["LC_label"]

# Handle missing values
if X.isnull().sum().sum() > 0:
    print("Warning: Missing values detected. Filling with median.")
    X.fillna(X.median(), inplace=True)

# === 2. APPLY FEATURE WEIGHTING ===
ev_weight = 3.0
serum_weight = 1.0
X[ev_markers] *= ev_weight
X[serological_markers] *= serum_weight

scaler = StandardScaler()
X_scaled = scaler.fit_transform(X)

# === 3. REPEATED EVALUATION (10× SPLITS) ===
acc2, prec2, rec2, f12 = [], [], [], []

for i in range(10):
    X_train, X_test, y_train, y_test = train_test_split(
        X_scaled, y, test_size=0.3, stratify=y, random_state=i
    )
    model = RandomForestClassifier(n_estimators=200, class_weight="balanced",
random_state=i)
    model.fit(X_train, y_train)
    y_pred = model.predict(X_test)

    acc2.append(accuracy_score(y_test, y_pred))
    prec2.append(precision_score(y_test, y_pred))
    rec2.append(recall_score(y_test, y_pred))
    f12.append(f1_score(y_test, y_pred))

print("\n=== Step 2 (LC vs. Non-LC) - 10× Evaluation ===")
print(f"Accuracy:  {np.mean(acc2):.4f} ± {np.std(acc2):.4f}")
print(f"Precision: {np.mean(prec2):.4f} ± {np.std(prec2):.4f}")
print(f"Recall:    {np.mean(rec2):.4f} ± {np.std(rec2):.4f}")
print(f"F1-score:  {np.mean(f12):.4f} ± {np.std(f12):.4f}")

# === 4. SINGLE RUN VISUALIZATION (random_state=42) ===
print("\n=== Single-Run Visualization for random_state=42 ===")
X_train, X_test, y_train, y_test = train_test_split(
    X_scaled, y, test_size=0.3, stratify=y, random_state=42
)
vis_model = RandomForestClassifier(n_estimators=200, class_weight="balanced",
random_state=42)
vis_model.fit(X_train, y_train)
```

```
y_pred_vis = vis_model.predict(X_test)

print("\nClassification Report (random_state=42):")
print(classification_report(y_test, y_pred_vis))

cm = confusion_matrix(y_test, y_pred_vis)
plt.figure(figsize=(6,5))
sns.heatmap(cm, annot=True, fmt="d", cmap="Blues",
            xticklabels=["Non-LC", "LC"], yticklabels=["Non-LC", "LC"])
plt.xlabel("Predicted")
plt.ylabel("True")
plt.title("Confusion Matrix - Step 2 (LC vs. Non-LC) [random_state=42]")
plt.tight_layout()
plt.show()
```

Python Decision Tree Code LC vs. Non-LC

```
# === IMPORT LIBRARIES ===
# === IMPORT LIBRARIES ===
import pandas as pd
import numpy as np

from sklearn.tree import DecisionTreeClassifier
from sklearn.model_selection import train_test_split, cross_val_score, StratifiedKFold
from sklearn.preprocessing import StandardScaler

# === LOAD & CLEAN DATA ===
df = pd.read_csv("Data_4_Group4_Filled_From_Group3_FIXED_Final.csv")
df = df[df["Group"] != "Group5: OTHER CANCER"]

# === DEFINE FEATURES & LABEL ===
features = ["EpCAM/CD133/gp38", "AFP", "CEA", "CA19-9", "Bilirubin"]
X = df[features].copy()
y = df["LC_label"].astype(int)

# === PREPROCESS ===
X.fillna(X.median(), inplace=True)
X["EpCAM/CD133/gp38"] *= 3.0
scaler = StandardScaler()
X_scaled = scaler.fit_transform(X)

# === EVALUATION FUNCTION ===
def evaluate_split(X_data, y_data, strategy_name):
    scores = {"Evaluation": [], "Metric": [], "Mean": [], "SD": []}
    model = DecisionTreeClassifier(max_depth=4, class_weight="balanced", random_state=42)
    cv = StratifiedKFold(n_splits=10, shuffle=True, random_state=42)

    for metric in ["accuracy", "precision", "recall", "f1"]:
        result = cross_val_score(model, X_data, y_data, cv=cv, scoring=metric)
        scores["Evaluation"].append(strategy_name)
        scores["Metric"].append("F1-score" if metric == "f1" else metric.capitalize())
        scores["Mean"].append(np.mean(result))
        scores["SD"].append(np.std(result))
    return pd.DataFrame(scores)

# === RUN FOR EACH STRATEGY ===
splits = {
    "50/50": 0.5,
    "60/40": 0.4,
    "70/30": 0.3,
```

```
    "80/20": 0.2,
    "90/10": 0.1,
    "Full Fit": 0.0,
}

all_results = []

for label, test_size in splits.items():
    if test_size > 0:
        X_train, _, y_train, _ = train_test_split(X_scaled, y, test_size=test_size,
stratify=y, random_state=42)
    else:
        X_train, y_train = X_scaled, y
    all_results.append(evaluate_split(X_train, y_train, label))

df_summary = pd.concat(all_results, ignore_index=True)

# === SPLIT DATA ===
from sklearn.model_selection import train_test_split
from sklearn.tree import DecisionTreeClassifier
from sklearn.metrics import confusion_matrix, ConfusionMatrixDisplay

X_train, X_test, y_train, y_test = train_test_split(X_scaled, y, test_size=0.3,
stratify=y, random_state=42)

# === TRAIN MODEL ===
model = DecisionTreeClassifier(max_depth=4, class_weight="balanced", random_state=42)
model.fit(X_train, y_train)

# === VISUALIZE DECISION TREE (70/30 Split) ===
from sklearn.tree import plot_tree

plt.figure(figsize=(20, 10))
plot_tree(
    model,
    feature_names=features,
    class_names=["Non-LC", "LC"],
    filled=True,
    rounded=True,
    impurity=True,
    fontsize=10
)
plt.title("Decision Tree – 70/30 Train-Test Split (random_state=42)", fontsize=16)
plt.tight_layout()
plt.savefig("DecisionTree_70_30_Split_Colored.png", dpi=300)
plt.show()


# === PREDICT & CONFUSION MATRIX ===
y_pred = model.predict(X_test)
cm = confusion_matrix(y_test, y_pred)
TN, FP, FN, TP = cm.ravel()

# === CALCULATE SENSITIVITY & SPECIFICITY ===
sensitivity = TP / (TP + FN)
specificity = TN / (TN + FP)

print("=== 70/30 Confusion Matrix Metrics ===")
print(f"Sensitivity (Recall):  {sensitivity:.3f}")
print(f"Specificity:           {specificity:.3f}")

# === PLOT CONFUSION MATRIX ===
```

```
import matplotlib.pyplot as plt
import seaborn as sns

plt.figure(figsize=(6,5))
sns.heatmap(cm, annot=True, fmt="d", cmap="Blues",
            xticklabels=["Non-LC", "LC"], yticklabels=["Non-LC", "LC"])
plt.xlabel("Predicted")
plt.ylabel("True")v
plt.title("Confusion Matrix - DT (70/30 Split, random_state=42)")
plt.tight_layout()
plt.show()

from sklearn.metrics import roc_auc_score

# === GET PROBABILITIES FOR AUROC ===
y_proba = model.predict_proba(X_test)[:, 1]  # Probabilities for the positive class

# === CALCULATE AUROC ===

auroc = roc_auc_score(y_test, y_proba)

print(f"AUROC (70/30 Split):   {auroc:.3f}")

# === EXPORT TO CSV (OPTIONAL) ===
df_summary.to_csv("DT_10xCV_Transparency_Table.csv", index=False)

# === DISPLAY RESULTS ===
df_summary
```
